# Genetic visualization of protein interactions harnessing liquid phase transitions

**DOI:** 10.1038/srep46380

**Published:** 2017-04-13

**Authors:** Taku Watanabe, Tatsuya Seki, Takashi Fukano, Asako Sakaue-Sawano, Satoshi Karasawa, Misaki Kubota, Hiroshi Kurokawa, Ken Inoue, Junichi Akatsuka, Atsushi Miyawaki

**Affiliations:** 1 Laboratory for Cell Function and Dynamics, Brain Science Institute, RIKEN, Saitama 351-0198, Japan; 2 Ina Laboratory, Medical & Biological Laboratories Co., Ltd., Ina, Nagano, Japan; 3 Amalgaam Co. Ltd., Ina, Nagano, Japan; 4 Biotechnological Optics Research Team, Center for Advanced Photonics, RIKEN, Saitama 351-0198, Japan

## Abstract

Protein-protein interactions (PPIs) are essential components of cellular function. Current fluorescence-based technologies to measure PPIs have limited dynamic range and quantitative reproducibility. Here, we describe a genetically-encoded PPI visualization system that harnesses the dynamics of condensed liquid-phase transitions to analyze protein interactions in living cells. The fluorescent protein Azami-Green and p62-PB1 domain when fused to PPI partners triggered a rapid concatenation/oligomerization process that drove the condensation of liquid-phase droplets for real-time analysis of the interaction with unlimited dynamic range in the fluorescence signal. Proof-of-principle studies revealed novel insights on the live cell dynamics of XIAP-Smac and ERK2-dimer interactions. A photoconvertible variant allowed time-resolved optical highlighting for PPI kinetic analysis. Our system, called Fluoppi, demonstrates the unique signal amplification properties of liquid-phase condensation to detect PPIs. The findings introduce a general method for discovery of novel PPIs and modulators of established PPIs.

Protein-protein interactions (PPIs) are fundamental components of cell structure and function. PPIs also are essential for disease pathogenesis and represent important targets for drug discovery^1^. Finding new drugs (compounds) that modulate disease-related PPIs is an important goal of biomedical research, and various fluorescent protein (FP)-based imaging methods that monitor PPIs have been developed and practically used, including fluorescence resonance energy transfer (FRET)^2^, bimolecular fluorescence complementation (BiFC)^3^, and dimerization-dependent FP (ddFP)^4^. All of these current methods detect the co-proximity of fusion protein tags in particular atomic orientations and thus require painstaking optimization of fusion constructs. Also, each of these methods have significant limitations. FRET monitors PPIs in real time but its limitations include a very low dynamic range, and significant effort and expertise for its system design, operation, and data analysis. BiFC visualizes PPIs with high sensitivity, but exhibits a substantial latent period and is irreversible, making *in vivo* assessment of PPIs difficult. ddFP shows a moderate dynamic range with substantial background signal, although it has recently evolved to function as ratiometric biosensors^5^.

Another well-established method that monitors PPI-dependent translocation of an FP-tagged protein to an artificially specified subcellular site (platform) is the fluorescent two- and three-hybrid systems^6^,^7^. These methods are gaining popularity in the area of high-content analysis (HCA) because they are simple and compatible with recent advances in hardware and software^8^. However, the signal occurrence on a chromatin site, for example, is not sufficiently distinct to allow easy detection.

A recently developed PPI detection method called InCell SMART-i utilizes the cluster formation of multivalent particles through efficient crosslinking^9^; ferritin nanoparticles labeled with FPs are assembled in a PPI-dependent manner to form intracellular clusters recognizable as fluorescent dots. Whereas several molecular associations have been studied by this method^10^, no molecular dissociation has been well documented. It is likely that the clusters are entangled aggregates in which crosslinked products do not diffuse freely. Furthermore, a critical component of this technology, rapamycin, which is utilized for conjugating target proteins to pre-formed nanoparticles, inhibits the activities of mTOR complexes^11^, potentially leading to the modulation of many cellular functions, such as protein synthesis, lipid synthesis, autophagy, lysosome biogenesis, energy metabolism, and cell survival^12^ during PPI observation.

We sought to create a PPI detection method based on a qualitatively different physico-chemical principle that can overcome the limitations of existing methods. To achieve this goal, we examined the biochemical process underlying phase transitions of proteins into condensed liquid compartments, which is emerging as a ubiquitous process in intracellular organization where an increasing number of membrane-less intracellular structures behave like liquid-phase droplets in the cytoplasm/nucleoplasm^13^,^14^. While many of the structures are ribonucleoprotein (RNP) granules, such as P granules and nucleoli, a recent paper by Li *et al*. demonstrated that multivalent binding of signaling proteins can assemble liquid-phase droplets^15^. In the present study, we engineered two protein modules that have the capability to homo-oligomerize, to examine if a PPI-dependent association between the two modules drives phase transitions that promote the condensation of the crosslinked products into liquid-phase droplets. One protein module is the Phox and Bem1p (PB1) domain of p62/SQSTM1. The p62 PB1 domain contains conserved acidic/hydrophobic residues on one side and conserved lysine and arginine residues on the other side, and thus can self-interact in a front-to-back topology to form high-molecular-weight homo-oligomers^16^,^17^ (Fig. 1a, top). The second module is Azami-Green (AG), a coral-derived green-emitting FP that forms an obligate tetrameric complex^18^ (Fig. 1a, bottom). Remarkably, using this system we detected PPI-dependent synthesis of liquid-phase droplets as the emergence of fluorescent puncta in living cells. The method, called Fluoppi, is amenable to current FP technologies and applicable to many different PPI monitoring systems that use HCA and high-throughput screening (HTS).

## Results

### Formation of cytoplasmic puncta by PB1 and Azami-Green (AG) fusion

**Figure 1:**
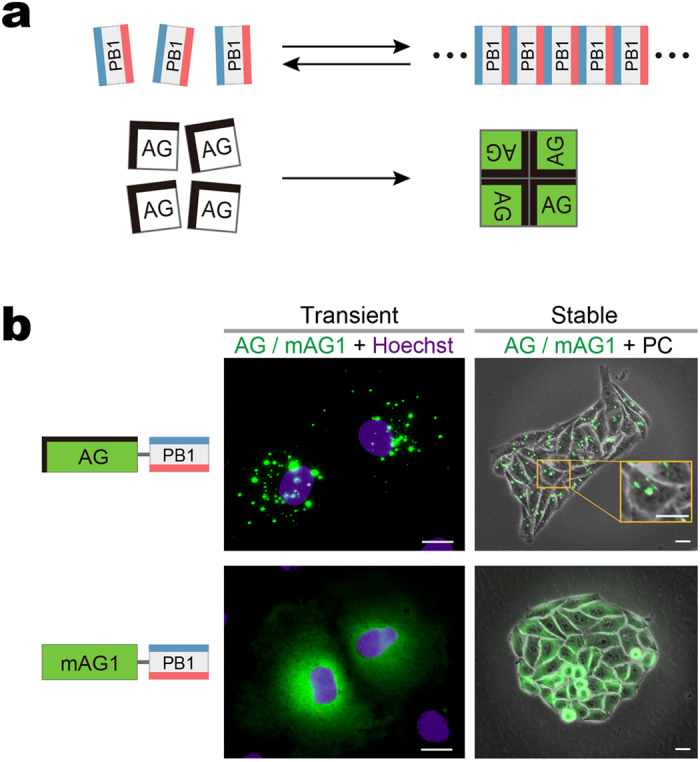
Cytosolic formation of fluorescent puncta composed of PB1 domain fused to AG. (**a**) Homo-oligomerization of the p62 PB1 domain (top) and the green-emitting fluorescent protein AG (Azami-Green) (bottom). *top*, PB1 self-associates in an equilibrium in a front-to-back topology to form a high-molecular-weight homo-oligomer. The conserved acidic/hydrophobic and lysine/arginine residues of PB1 are indicated by red and blue bars, respectively. *bottom,* AG forms an obligate tetramer complex to become fluorescent (green). The hydrophobic interfaces between AG subunits are indicated by thick bars on two adjacent sides. (**b**) Fluorescence images of cultured cells expressing PB1 fusion to AG and the monomeric mutant of AG (mAG1), AG-PB1 and mAG1-PB1, respectively. The absence of a thick black bar indicates that mAG1 has no hydrophobic patches on its surface. (AG/mAG1 + Hoechst) Fluorescence images of Cos-7 cells transiently expressing AG-PB1 (upper) and mAG1-PB1 (lower) one day post-transfection. Images of AG/mAG1 (green) and Hoechst33342-stained nuclei (violet) were merged. The same distribution pattern was observed in approximately 50 other cells for each construct. (AG/mAG1 + PC) Fluorescence images of HeLa cells stably expressing AG-PB1 (upper) and mAG1-PB1 (lower). Their phase-contrast (PC) images were merged. A high-magnification image is shown to zoom in on fluorescent clusters of AG-PB1 (orange inset). The same distribution pattern was observed in 4 other HeLa cell clones for each construct. Scale bars, 5 μm. See also Supplementary Fig. 1.

As revealed by *in vitro* studies^16^,^17^, electrostatic interactions between adjacent acidic and basic surfaces of the p62 PB1 domain leads to homo-oligomerization, forming a high-molecular-weight product. It was also found that the PB1 domain was critical for the formation of cytoplasmic bodies (punctate structures) in cells transfected with p62-derived constructs^19^. While this *in vivo* finding was obtained from experiments using GFP-tagged constructs, it was noted that any tag would pose a risk to the construct by affecting its behavior. Therefore, we transfected various FP fusions of PB1 into HeLa cells and examined their intracellular distribution. A remarkable difference was observed regarding the fluorescence distribution in the cytosol when PB1 was fused to AG and its monomeric mutant (mAG1)^18^ (Fig. 1b, Transient). Whereas cells (Cos-7) expressing AG-PB1 exhibited bright micrometer-sized fluorescent puncta, cells expressing mAG1-PB1 showed a diffuse pattern. Such a differential distribution was observed also in HeLa cells stably expressing AG-PB1 and mAG1-PB1 (Fig. 1b, Stable), suggesting sustainability of the two extremes. The same result was obtained from transient expression experiments that used other cell types (Supplementary Fig. 1a,b). Since AG is coral-derived and resistant to lysosomal proteases, the fluorescent puncta might reflect lysosomes accumulating AG-PB1 as a result of basal levels of autophagy^20^. However, our confocal microscopy observation showed that the fluorescent puncta of AG-PB1 were not colocalized with lysosomes (Supplementary Fig. 1c). These results suggest that whereas mAG1-PB1 freely diffuses in the cytoplasm, AG-PB1 is condensed into visible cytoplasmic bodies. Remarkably, the punctum formation by AG-PB1 transfection was constantly observed irrespective of the confluence and cell cycle of recipient cells (data not shown). In addition, the AG-PB1 stable transformant cells showed the same proliferation rate as control cells (data not shown). Based on these results, we expected that the observed fluorescent puncta could serve as a reliable readout of the quaternary structure of FP fusion constructs in living cells.

### Visualizing punctum formation by PPI-dependent association of PB1 and AG

**Figure 2:**
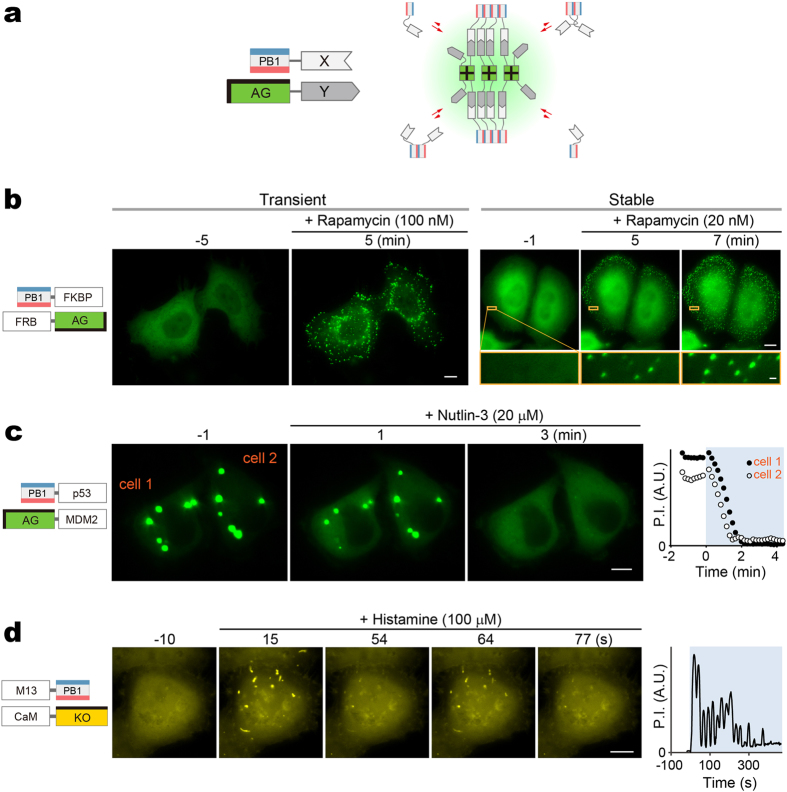
Design and validation of Fluoppi, a PPI detection system using PB1/AG tags. (**a**) Schematic representation of PPI-dependent formation of fluorescent puncta. Due to the interaction between X and Y, PB1-X and AG-Y build crosslinks, resulting in the concentration of AG fluorescence (green shading). PB1 and AG are depicted as shown in Fig. 1. (**b**) Visualization of rapamycin-induced association between FRB and FKBP in HeLa cells co-expressing PB1-FKBP and FRB-AG transiently (left) and stably (right). To show the size of puncta, a part of the cytosol (orange box) is magnified. See also Supplementary Videos 1 and 2. The same results were obtained from approximately 50 cells in both transient and stable experiments. (**c**) Visualization of nutlin-3-induced dissociation of p53/MDM2 complex in two HeLa cells (cell 1 and cell 2) that transiently co-expressed PB1-p53 and AG-MDM2. See also Supplementary Video 3. The same results were obtained from approximately 50 cells in 3 independent experiments. (**d**) Visualization of histamine-induced oscillatory association between CaM and M13 in a HeLa cell that co-expressed M13-PB1 and CaM-KO. The image acquisition interval was 1.5 seconds. KO forms an obligate dimeric complex. The hydrophobic interface between KO monomers is indicated by a thick bar on one side. See also Supplementary Video 5. Similar oscillatory changes were observed in 4 other cells. (**b**–**d**) Domain structures of transfected constructs are illustrated (leftmost). Each domain is depicted by a rectangle. The time point of image acquisition relative to drug administration is indicated above each image. (**c**,**d**) Total fluorescence intensity of cytosolic puncta in a cell (punctum intensity, P.I.) was plotted against time (rightmost). Scale bars, 10 μm. Scale bar in magnified box (**b**), 1 μm. See also Supplementary Fig. 2.

We examined if PPIs between two given target domains (X and Y) could be observed by fusing their respective interaction domains to AG and PB1 and detecting the formation of fluorescent puncta in living cells (Fig. 2a). The feasibility of this methodology was tested by using the FKBP12 (FKBP) and FRB domain of mTOR as X and Y, respectively^21^,^22^ (Fig. 2b). After co-transfection of PB1-FKBP and FRB-AG into HeLa cells (Fig. 2b, Transient), a homogenous distribution of green fluorescence was observed throughout the cytosol at first (−5 min). However, after treatment with 100 nM rapamycin for several minutes, the fluorescence distribution dramatically changed to form dozens of submicrometer-sized puncta (5 min). The kinetics of punctum formation was studied by time-lapse imaging using HeLa cells stably co-expressing PB1-FKBP and FRB-AG (Fig. 2b, Stable, Supplementary Video 1). A few minutes after the addition of 20 nM rapamycin, submicrometer-sized fluorescent puncta became detectable in the cytosol. When following closely for a longer time period (~70 min), we observed their frequent fusion into larger objects (Supplementary Video 2). In most cases, two puncta fused upon touching and relaxed their shape in a few minutes (Supplementary Fig. 2a), suggesting that the observed punctate structures are condensed liquid-like phases.

These results indicate that not only covalent but also non-covalent ligation between AG and PB1 can form identifiable fluorescent puncta presumably based on the principle of their multivalency; both AG and PB1 show homo-oligomerizing properties and their ligation results in crosslinking for condensation. In fact, when AG was replaced with mAG1 or when PB1 was mutated to suppress its oligomerization (PB1(D67A/D69R)), rapamycin (100 nM) treatment did not change the intracellular fluorescence distribution from homogenous to punctate patterns (Supplementary Fig. 2b).

### Reversible, rapid, and ubiquitous PPI detection using PB1/AG system

Next, we asked whether the fluorescent puncta were dissociable. To examine the reversibility of the AG fluorescence distribution between homogenous and punctate patterns, we employed p53 and MDM2 whose molecular association can be blocked by the administration of a specific PPI inhibitor, nutlin-3 (ref. 23). After PB1-p53 and AG-MDM2 were transiently co-transfected into HeLa cells, many submicrometer-sized clusters emerged, which later grew in size via fusion. One day post-transfection, several clear fluorescent puncta having diameters of 1–5 μm appeared in the cytosol (Fig. 2c, −1 min). To quantitate these punctate patterns, we measured the total fluorescence of identifiable puncta within a cell. The value “punctum intensity (P.I.)” could be measured on fluorescence images (Supplementary Fig. 2c) and was used as an index of PPI in the present study. Upon administration of nutlin-3 (20 μM), the puncta began to melt (Fig. 2c, 1 min) and then completely disappeared within three minutes (Fig. 2c, 3 min, Supplementary Video 3). The P.I. values of cells 1 and 2 were plotted against time (Fig. 2C, rightmost). The fluorescent punctate structures were different from lysosomes (Supplementary Fig. 1d). It was concluded that a reversible PPI between the two protein domains can be visualized in a live cell by genetically fusing them to PB1 and AG. Importantly, the PB1/AG system can be applied to PPIs that take place not only inside the cytosol but also in the nucleus (Supplementary Fig. 2d) and beneath the plasma membrane (Supplementary Fig. 2e, Video 4). We termed this method Fluoppi for Fluorescent ppi-visualization. Fluoppi was previously employed as a general method for PPI detection^24^,^25^,^26^ before its operational principle was understood or studied.

We examined if AG can be replaced with other oligomeric FPs. We used an orange-emitting FP, Kusabira-Orange (KO), which exists mostly in dimers^27^. Using Fluoppi with KO, we monitored in real time the calcium-dependent interactions between calmodulin (CaM) and its target peptide (M13)^28^ (Fig. 2d). M13-PB1 and CaM-KO were co-transfected into HeLa cells, which were then subjected to a time-lapse imaging experiment with a sampling interval of 1.5 sec. A supra-maximal dose of histamine (100 μM) caused the oscillatory formation of approximately 10 micropuncta per cell for over several minutes (Supplementary Video 5). Interestingly, the size and location of each punctum were constant^29^. We plotted P.I. measured in the observed cell against time and obtained an oscillatory pattern (Fig. 2d, rightmost) that was similar to typical calcium transients^28^. These results suggest that Fluoppi can monitor reversible PPIs repetitively on a time scale of seconds, and that it is possible to expand the repertoire of FPs for the design of multi-color Fluoppi for multiple PPI detections.

### Application to drug screening assays with low background and high reliability

**Figure 3:**
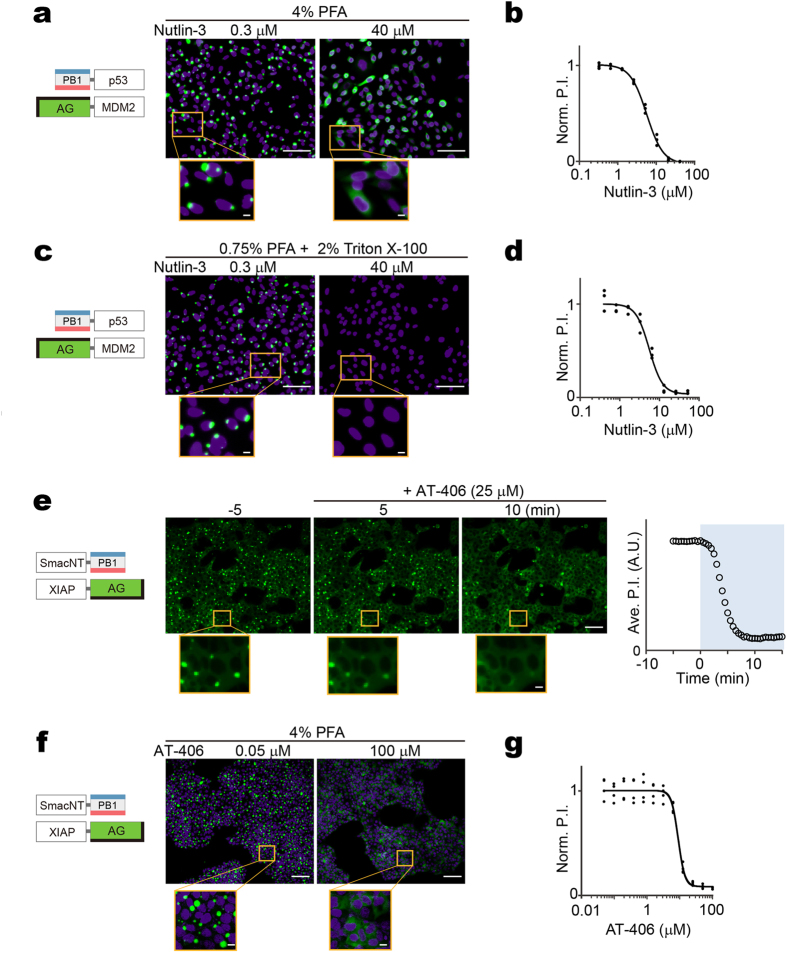
Application of Fluoppi to quantitative analysis of drug-induced PPI blockage. (**a**,**b**) High-content analysis (HCA) approach to the quantification of nutlin-3-induced dissociation of p53/MDM2 complex in CHO-K1 cells stably co-expressing PB1-p53 and AG-MDM2. After treatment of various concentrations of nutlin-3 for 30 min, cells were fixed with 4% PFA for 10 min and their nuclei were stained with Hoechst33342. (**a**) Domain structures of transfected constructs and two representative images of cells treated with 0.3 and 40 μM nutlin-3. (**b**) Dose (nutlin-3 concentration)-response (normalized P.I.) curve. Both puncta and nuclei were segmented automatically. P.I. was obtained by dividing the total AG (green) fluorescence from all the puncta by the total number of nuclei in each field of view. Normalized to the P.I. value at the lowest concentration.(**c**,**d**) High-throughput screening (HTS) approach to the quantification of nutlin-3-induced dissociation of p53/MDM2 complex in CHO-K1 cells stably co-expressing PB1-p53 and AG-MDM2. After treatment of various concentrations of nutlin-3 for 30 min, cells were fixed and permeabilized with PBS(−) containing 0.75% PFA and 2% Triton X-100 for 10 min and their nuclei were stained with Hoechst33342. (**c**) Domain structures of transfected constructs and two representative images of cells treated with 0.3 and 40 μM nutlin-3. (**d**) A dose (nutlin-3 concentration)-response (normalized P.I.) curve. No segmentation was performed. P.I. was obtained by dividing the total AG (green) fluorescence by the total Hoechst33342 (blue) fluorescence for each field. Normalized to the P.I. value at the lowest concentration. (**e**–**g**) Quantification of AT-406-induced dissociation of Smac/XIAP complex in HEK293 cells stably co-expressing SmacNT-PB1 and XIAP-AG. (**e**) Domain structures of transfected constructs are depicted (leftmost). Fluorescence images of cells 5 min before and 5 and 10 min after the addition of 25 μM AT-406 are shown (middle). Puncta were segmented automatically, and cell number was counted manually. The time course of averaged P.I. is shown (rightmost). See also Supplementary Video 6. (**f**) Domain structures of transfected constructs and two representative images of cells treated with 0.05 and 100 μM AT-406. (**g**) Dose (AT-406 concentration)-response (normalized P.I.) curve. Both puncta and nuclei (Hoechst33342-stained) were segmented automatically. P.I. was obtained by dividing the total AG (green) fluorescence from all the puncta by the total number of nuclei in each field of view. Normalized to the P.I. value at the lowest concentration. (**a**–**g**) Each experiment was performed in triplicate. Scale bars, 100 μm. Scale bars in magnified boxes, 10 μm. See also Supplementary Fig. 3.

We next examined whether Fluoppi could be used in HCAs to discover and characterize compounds that block PPIs (PPI blockers). We found CHO-K1 cells stably expressing both PB1-p53 and AG-MDM2 each carried a few gigantic fluorescent puncta that disappeared immediately after the addition of nutlin-3 as shown in the transient experiment (Fig. 2c). Remarkably, these punctate structures were completely resistant to conventional fixation procedures, such as treatment with 4% paraformaldehyde (PFA) for ten minutes. Accordingly, the fluorescent puncta were quantifiable in fixed cell samples. This feature suggests that Fluoppi can be a robust technique suitable for drug screening assays that usually analyze signals at end time points. Indeed, a significant difference in fluorescence distribution was observed after the addition of 0.3 and 40 μM nutlin-3 and subsequent fixation (Fig. 3a). We quantified the extent of the PPI between PB1-p53 and AG-MDM2 by measuring the P.I. with various concentrations of nutlin-3 (Fig. 3b). The IC50 value was 5.7 ± 0.6 μM (mean ± SD, n = 3), consistent with that determined by previous cell-based assays^8^. Similarly, the EC50 value of rapamycin for FRB-AG/PB1-FKBP was calculated to be 15.1 ± 1.7 nM (Supplementary Fig. 3a,b), which was also in the correct range of affinity as determined previously^30^.

The differential diffusion properties of AG between the two extreme distributions of Fluoppi would make it possible to create a HTS system for the quantification of PPIs. When 2% Triton X-100 was added to permeabilize the plasma membrane during fixation with 0.75% PFA, we observed that diffusible AG-containing molecules were washed off the cells whereas AG-containing punctate structures were well retained inside (Fig. 3c). Thus, after washing, the measurement of the remaining fluorescence with a photomultiplier tube (PMT) can provide a quantitative readout of PPIs. To demonstrate a proof of principle for this HTS application, we studied the dose-response relationship of nutlin-3 for PB1-p53/AG-MDM2 using a microplate reader. In this situation, the total AG fluorescence divided by the Hoechst fluorescence in a given field could be plotted as an equivalent to P.I. We obtained an IC50 value of 5.4 ± 0.4 μM (mean ± SD, n = 3) (Fig. 3d), which was similar to established values (5.7 μM) (Fig. 3b).

We further explored whether Fluoppi provides a platform for identifying small-molecule inhibitors of the X-linked inhibitor of apoptosis protein (XIAP). Anti-apoptotic proteins, including those of the inhibitor of apoptosis protein (IAP) family, play important roles in chemoresistance, and XIAP is the best known IAP member. XIAP has received significant attention as a drug target; its inhibition primes cancer cells for death induced by cytotoxic agents^31^. As Smac (second mitochondria-derived activator of caspase) is an endogenous antagonist of XIAP, PPI between XIAP and Smac can be used to design a platform to search for small-molecule Smac mimics. We applied the BIR3 domain of XIAP and the N-terminal region of Smac (SmacNT) to create a Fluoppi assay, and prepared HEK293 cells that stably co-expressed SmacNT-PB1 and XIAP-AG. These cells each carried a few large fluorescent puncta inside the cytosol (Fig. 3e, −5 min). Then, we added the XIAP inhibitor, AT-406, which sensitizes the response of ovarian cancer cells to carboplatin^32^. Time-lapse imaging revealed that the cytosolic puncta disappeared within ten minutes after the addition of 25 μM AT-406 (Fig. 3e, Supplementary Video 6). HCA experiments with automatic segmentation of puncta and nuclei (Fig. 3f) revealed that AT-406 inhibited PPI between BIR3 (XIAP) and Smac (SmacNT) with an IC50 value of 9.2 ± 1.2 μM (mean ± SD, n = 4) (Fig. 3g), consistent with values observed from cytotoxicity assays^33^. Also, the PPI modulating performance of AT-406 (50 μM) was compared by time-lapse imaging with that of another well-known XIAP inhibitor, LCL-161 (50 μM) (Supplementary Fig. 3c,d). AT-406 had a slower but more potent and persistent effect on PPIs than LCL-161.

### Photoconvertible Fluoppi variation for studying PPI kinetics

As Fluoppi can monitor reversible PPI, we equipped the Fluoppi system with an optical highlighting technology for time-resolved analysis. Substitution of a tetrameric photoconvertible FP for AG allowed us to examine the exchange rate of the FP between highlighted and non-highlighted puncta in a living cell. We cloned the cDNA encoding a new photoconvertible FP Momiji (Mmj) from *Scolymia vitiensis*. Mmj shows 74% amino acid sequence homology to Kaede with the conserved tripeptide (His63-Tyr64-Gly65) for chromophore formation^34^ (Supplementary Fig. 4a). Like Kaede, Mmj is a green-emitting FP that can be converted into a red emitter by (ultra-)violet light irradiation (Supplementary Fig. 4b–e). However, Mmj showed brighter fluorescence and a higher photoconversion quantum efficiency than Kaede (data not shown). Since Mmj is an obligate tetramer, it can also be used in the Fluoppi system.

**Figure 4:**
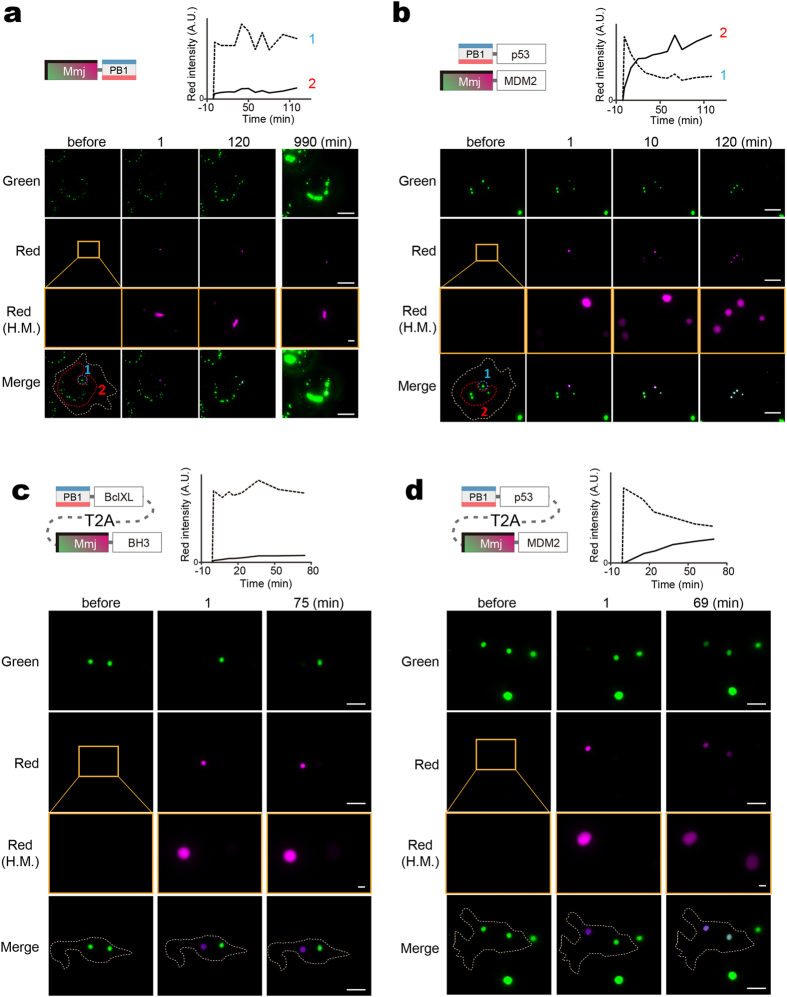
PcFluoppi using PB1 and Mmj for analysis of PPI kinetics. Hydrophobic interfaces between Mmj subunits are indicated by thick bars on two adjacent sides. (**a**,**b**) Inter-punctum exchange of Mmj fluorescence in Cos-7 cells (outlined by white dotted lines) after local UV light irradiation. The photoconverted (red) fluorescence was measured in the UV-irradiated regions (1, encircled by blue dotted lines) and the intact regions (2, encircled by red dotted lines), and their intensities were plotted against time (top). (**a**) One day post-transfection of Mmj-PB1, cells were imaged for green and red fluorescence before and 1, 120, and 990 min after photoconversion. (**b**) One day post-cotransfection of PB1-p53 and Mmj-MDM2, cells were imaged for green and red fluorescence before and 1, 10, and 120 min after photoconversion. (**c**,**d**) Inter-punctum exchange of Mmj fluorescence in HEK293 cells (outlined by white dotted lines) that expressed PB1-BclXL:T2A:Mmj-BH3 (**c**) and PB1-p53:T2A:Mmj-MDM2 (**d**). In each experiment, a cell carrying two big puncta was chosen and one punctum was specifically UV irradiated. The intensities of red fluorescence from the UV-irradiated (dotted line) and intact (solid line) puncta were plotted against time (top). (**a**–**d**) Similar results were obtained from 3 other cells for each transfection. Scale bars, 10 μm. Scale bars in high-magnification (H.M.) boxes, 1 μm. See also Supplementary Fig. 4.

We first transfected Mmj-PB1 into Cos-7 cells (Fig. 4a). One day post-transfection, approximately ten green fluorescent puncta were identified in each transfected cell, and one of them was photoconverted by local irradiation of ultraviolet (UV) light. We found that the red and green signal intensities were stable during the 120- minute observation period. Even at 990 minutes post-photoconversion, until the time when newly synthesized green signals had been added to surpass the signals from the red puncta, the original shape and intensity of the red signals were nearly perfectly preserved.

We next co-transfected PB1-p53 and Mmj-MDM2 into Cos-7 cells (Fig. 4b). Using a separate culture dish, we verified their reversible interaction by the administration of 20 μM nutlin-3 (Supplementary Fig. 4f), as performed for PB1-p53/AG-MDM2 (Fig. 2c). We chose a cell that carried four green puncta and photoconverted one of them. The red/green contrast was high after one minute, but gradually decreased. After two hours, all the four puncta were equally red. It was thus concluded that Mmj-MDM2 and possibly PB1-p53 as well were migrating among the four puncta. These results suggest that the inter-punctum exchange of Fluoppi constructs is largely dependent on the on/off rates of PPI between X and Y rather than the homo-oligomerizing kinetics of PB1; the tetrameric complex of Mmj (AG) is considerably tightly packed and non-dissociable^35^.

Finally, we used a photoconvertible Fluoppi (pcFluoppi) variant as a method to characterize PPI affinity. We focused on PPI between BclXL and the BH3 peptide from BAD. Their interaction was very strong as characterized by the low *K*_d_ value of 0.6 nM^36^. To perform a more quantitative analysis, we used the T2A bicistronic expression system to express PB1-BclXL and Mmj-BH3 with 1:1 stoichiometry. Likewise, we expressed equal amounts of PB1-p53 and Mmj-MDM2 as a weak control of PPI; a reported *K*_d_ value of MDM2/p53 was 260 nM^37^. In each experiment, we chose a cell possessing two green puncta. During the one-hour observation after the photoconversion of one punctum, the exchange of green and red signals between the two puncta was not seen for PB1-BclXL/Mmj-BH3 (Fig. 4c) but was substantially detected for PB1-p53/Mmj-MDM2 (Fig. 4d). Similar results were obtained from other three cells expressing each of the two combinations (Supplementary Fig. 4g,h).

### Demonstrating Fluoppi droplets behave as liquid-phase compartments

**Figure 5:**
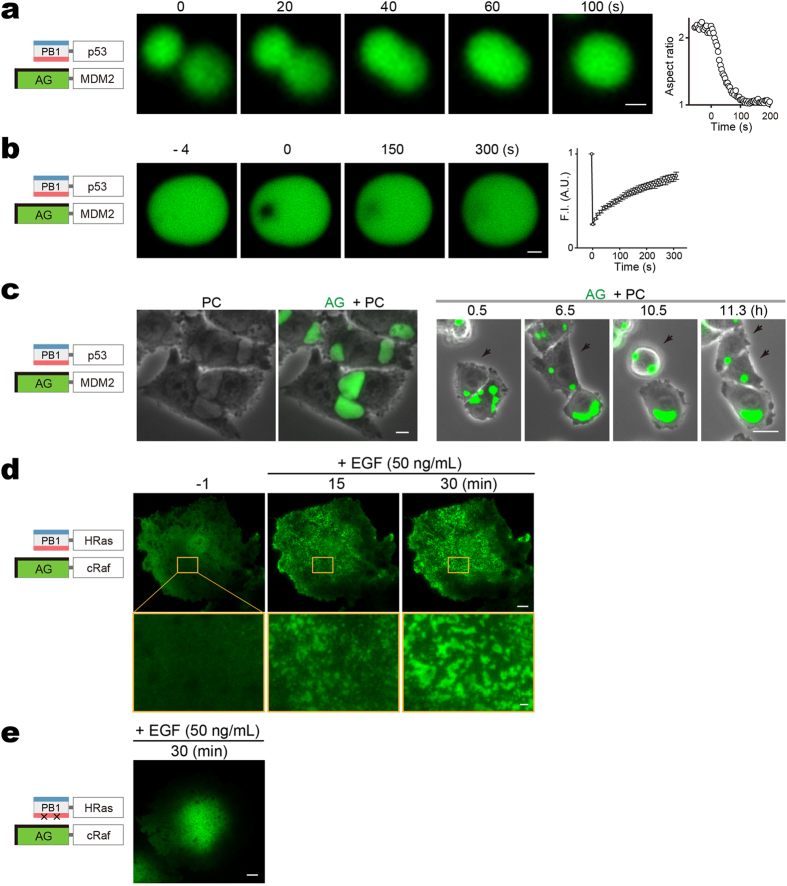
Liquid phase transitions for Fluoppi punctate structures. (**a**–**c**) Liquid-like state inside Fluoppi punctate structures in CHO-K1 cells stably co-expressing PB1-p53 and AG-MDM2. (**a**) Cells were treated with 20 μM nutlin-3 to cause punctum disappearance. After the drug was washed away, formation and growth of puncta were observed. Upon touching (0 s), two round puncta (~2 μm in diameter) fused and relaxed their shape in 2 minutes. The change in aspect ratio was plotted over time (rightmost). Similar results were obtained using 4 other puncta with diameters of <3 μm. (**b**) Application of FRAP to a round punctum (5 μm in diameter). The change in fluorescence intensity in the bleached region was plotted over time (rightmost). Similar results were obtained from 4 other puncta with diameters of <10 μm. (**c**) Cells were time-lapse imaged. PC, phase-contrast images. AG + PC, fluorescence images merged with phase-contrast images. *right*, A montage of a time-lapse imaging (AG + PC) of proliferating cells. One cell-division event is marked by black arrows. See also Supplementary Video 7. Most large puncta (>10 μm) were irregular in shape. Similar images were obtained using another CHO-K1 clone. (**d**,**e**) TIRF microscopy images. 2D phase separation (spinodal decomposition) was seen on the surface of a Cos-7 cell co-expressing PB1-HRas and AG-cRaf after stimulation with EGF (C). The same pattern was observed in 5 other cells. This pattern was not seen when monomeric PB1 (mPB1) was used (See Figure S2B). See also Supplementary Video 8. (**a**–**d**) Domain structures of transfected constructs are illustrated (leftmost). Scale bars, 1 μm (**a**,**b**); 10 μm (**c**–**e**). Scale bar in magnified box (**d**), 1 μm.

We examined puncta of PB1-p53/AG-MDM2 in the stable transformants (CHO-K1) (Fig. 3) to demonstrate that their internal organization was dynamic and uniform. First, round puncta (1–6 μm in diameter) were used for fusion observation (Fig. 5a) and FRAP experiment (Fig. 5b); the relaxation and recovery times suggest a dynamic meso-scale organization within individual puncta. Next, we acquired high-resolution phase-contrast images of cells that carried large fluorescent puncta (>10 μm in size) (Fig. 5c, left, PC). When compared with fluorescence images (Fig. 5c, left, AG + PC), it became clear that the punctate structures consisted of a uniform medium indicative of a liquid phase. We carried out a time-lapse imaging experiment and observed that these well-grown puncta were deformable according to cell movement, and fissionable upon cell division (Fig. 5c, right, Supplementary Video 7).

Further evidence that Fluoppi puncta represent bonafide liquid-phase droplets was obtained from an experiment that measured co-assembly of PB1-HRas and AG-cRaf on the plasma membrane by total internal reflection fluorescence microscopy (Fig. 5d). EGF stimulation induced their association, and AG fluorescence transitioned to micron-sized clusters on the cell surface (Supplementary Video 8, left). This pattern was not observed when the concatemerization capability of PB1 was abolished (Fig. 5e, Supplementary Video 8, right). A similar 2D phase separation was observed by Banjade and Rosen on lipid bilayers of protein clusters containing the adhesion receptor Nephrin and its cytoplasmic partners Nck and N-WASP^38^.

### Visualizing homo-dimerization with single fusion constructs

**Figure 6:**
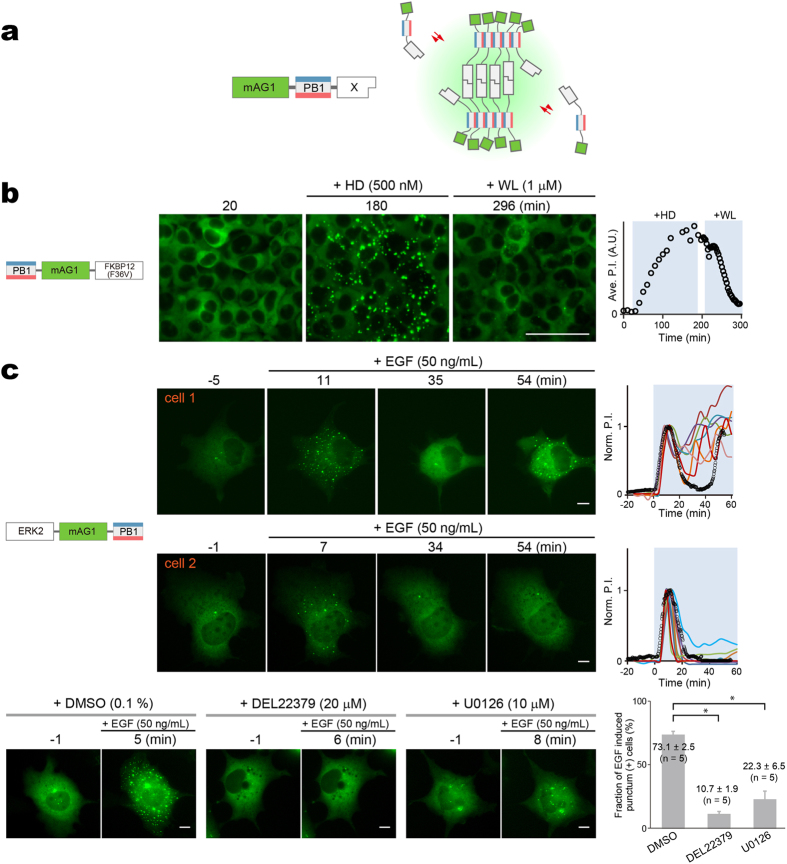
HomoFluoppi using mAG1-PB1 tag for visualizing homo-dimerization. (**a**) Schematic representation showing that due to the homo-dimerization of X, multiple mAG1-PB1-X molecules build crosslinks, resulting in the concentration of mAG1 fluorescence (green shading). (**b**) Pharmacologically regulated homo-dimerization of FKBP12(F36V) monitored in HEK293 cells that stably expressed PB1-mAG1-FKBP12(F36V). The homo-dimerization was induced by 500 nM HD and then blocked by 1 μM WL. Time points of image acquisition were counted after the observation was started. Puncta were segmented automatically, and cell number was counted manually. The time course of averaged P.I. is shown (rightmost). Scale bar, 100 μm. See also Supplementary Video 9. The experiment was performed in triplicate.(**c**) EGF-evoked homo-dimerization of ERK2 monitored in Cos-7 cells transiently expressing ERK2-mAG1-PB1. *top,* ERK2 homo-dimerization was quantified in 21 cells. Punctum formation was oscillatory in 9 cells and transient in 12 cells as represented by cell 1 (upper) and cell 2 (lower), respectively. Time points of image acquisition were counted after the addition of 50 ng/mL EGF. Puncta were segmented automatically. Despite heterogeneity of the temporal profile, initial punctum formation was observed 5–10 min after the addition of EGF. The time courses of P.I. normalized to the initial peak are shown (rightmost). Data points from cell 1 and cell 2 are indicated by black open circles. See also Supplementary Videos 10 and 11. *bottom*, Treatment of cells with DMSO, DEL22379, or U0126 for 30 min before the addition of 50 ng/mL EGF. Representative images are shown. Percentage of cells showing EGF-induced increase in P.I. in examined cells. Approximately 20 cells were observed in quintuplicate, and data are shown as mean ± s.e.m. Statistical significance (*p < 0.0001) was examined by Bonferoni’s multiple comparison test. Scale bars, 10 μm.

We extended the Fluoppi system to the visualization of protein homo-dimerization by developing a variant (homoFluoppi), in which a protein of interest (X) is simply fused to mAG1-PB1 (Fig. 6a). Homo-dimerization of X and homo-oligomerization of PB1 resulted in crosslinking to form liquid-phase droplets that emit green fluorescence. In this case, the use of AG is not appropriate because AG-PB1 always forms fluorescent puncta. To demonstrate the validity of homoFluoppi, we used an FKBP12 mutant with F36V mutation (FKBP12(F36V)). The homo-dimerization of FKBP12(F36V) can be well controlled pharmacologically; it is induced and subsequently disrupted by B/B Homodimerizer (HD) and B/B Washout Ligand (WL), respectively. We generated HEK293 cells stably expressing PB1-mAG1-FKBP12(F36V). Initially, the intracellular green fluorescence was homogenously distributed. After the addition of 500 nM HD, fluorescent puncta emerged and gradually grew large within a few hours. Then, after washing with PBS followed by the addition of 1 μM WL, the fluorescent puncta disappeared within one hour (Fig. 6b, Supplementary Video 9).

We evaluated the ability of Fluoppi to detect the homo-dimerization of ERK2 that has been shown *in vitro* but not consistently *in vivo* (Fig. 6c, Supplementary Videos 10 and 11). After transfection of ERK2-mAG1-PB1, cells exhibited only a few puncta in the cytosol. On EGF stimulation (50 ng/ml), both the number and size of puncta increased transiently peaking at approximately 10 min. In some cells, a second transient increase was observed later (Fig. 6c, cell 1). Such transient or pulsatile behavior was previously observed in nuclear translocation of ERK2 in EGF-stimulated cells individually^39^. The mAG1-fluorescent puncta were detected predominantly in the extra-nuclear space, which suggests that ERK homo-dimerization requires scaffold proteins in the cytosol^40^. To analyze the regulation of ERK2 homo-dimerization, we performed pharmacological experiments (Fig. 6c, bottom). First, we examined the effect of DEL22379, a small molecule inhibitor of ERK homo-dimerization^41^. After pretreatment with 20 μM DEL22379 for 30 min, the EGF-evoked increase in punctum formation was nearly blocked. Second, we pretreated transfected cells with the MEK inhibitor U0126 (10 μM). EGF-reactive punctum formation was significantly reduced suggesting that ERK homo-dimerization requires phosphorylation.

## Discussion

PPI monitoring methods are of great interest to researchers who avidly search for small-molecule mimics that modulate PPIs responsible for disease development. As *in vitro* methods may be contaminated by compounds having marked cell toxicity, *in vivo* fluorescence-based methods that monitor PPIs in living cells are required. Although the *in vivo* PB1/AG system is commercially available in the market as ‘Fluoppi’, we had not studied nor disclosed the mechanism of its operation principle. As Fluoppi is very simple and amenable to current FP technologies, it can be diversified into different modes for monitoring PPIs. Here, we discuss the theoretical principles and practical applications of Fluoppi and its variant applications called homoFluoppi and pcFluoppi, for the analysis of heteromeric PPIs, homomeric PPIs, and PPI kinetics, respectively (summarized in Supplementary Table 1).

Recently, non-membrane-bound compartments were shown to be comprised of liquid-phase droplets that arise by phase separation from the cytoplasm or nucleoplasm^13^,^14^. Judging from the rapid assembly/disassembly after the addition of regulators (Fig. 2, Supplementary Fig. 2a), the dynamic and uniform internal organization (Fig. 5a,b), the high deformability (Fig. 5c), and the capability to form spinodal decomposition on the plasma membrane (Fig. 5d), Fluoppi punctate structures have the hallmarks of condensed liquid-phase droplets. The operating mechanism of Fluoppi resides in a fusion protein complex containing two independent homo-oligomerizing modules; one of which is PB1 whose concatemers generate elastic crossbeams within the liquid-phase droplet. This basis of this result comes from previous reports that reveal phase transitions as a dynamic assembly of protein domains that are concatemeric as well as multivalent^15^,^42^. Li *et al*. showed that the co-expression of mCherry fused to a pentameric concatemer of the SH3 domain and EGFP fused to a pentameric concatemer of an SH3 ligand motif in HeLa cells resulted in the formation of 0.5–2-μm-diameter cytoplasmic puncta containing both FPs^15^. We speculate that the long and flexible PB1 concatemer is critical for the growth of liquid-phase droplets into large cellular bodies. Indeed, we did not see large puncta with any fusion protein (complex) that had two homo-oligomeric FPs but no PB1 (Supplementary Fig. 5). Li *et al*. also showed that increased valency of the interacting species significantly decreases their critical concentrations for the phase separation^15^. We suppose that Fluoppi, containing the high-molecular-weight homo-oligomers of PB1, achieves such multivalency that permits efficient formation of liquid droplets even at low expression levels.

Fluoppi offers several merits over existing FP-based PPI detection techniques (Supplementary Table 2). First, Fluoppi shows a large dynamic range: a PPI-dependent change in fluorescence distribution, likely due to the liquid-phase separation mechanism that concentrates molecules into liquid-phase droplets of unlimited capacity. In contrast, existing PPI detection methods that measure the translocation of FPs to preformed platforms suffer from a limited dynamic range and/or high background signal that often prohibits reliable measurement^6^,^7^. Therefore, conventional HCA systems that acquire and process image data will benefit from the robust response of Fluoppi for reproducible screening of drugs. Furthermore, Fluoppi signals are tolerant of fixation procedures and thus can be preserved for subsequent large-scale image analysis. We also showed that Fluoppi could be successfully combined with the HTS approach that did not use image data (Fig. 3c,d). Second, Fluoppi allows for rapid monitoring of protein-protein dissociation, which is useful for constructing platforms for the discovery of small molecules that inhibit PPIs. For example, we constructed an XIAP/Smac Fluoppi system to generate a reliable method for the discovery of small-molecule Smac mimics. We found that the drug-induced rapid disassembly of fluorescent puncta was mostly completed in half an hour as a result of phase transitions. By contrast, InCell SMART-i generates tough aggregates and principally detects protein-protein association events, but has not been reported for the detection of protein-protein dissociation, neither has NS viral inclusion body^43^. Third, Fluoppi is simple and easily applicable to various PPI systems, employing single FPs, unlike FRET and ddFP methods. In addition, Fluoppi requires only two gene constructs that can be concatenated through the T2A bicistronic expression system for transfection, and many types of cells are usable. By contrast, existing FP-translocation methods require recipient cells to have stable platforms formed in advance, and InCell SMART-i requires the preformation of ferritin nanoparticles. Lastly, Fluoppi can be diversified to create new PPI-monitoring methods by using other unique FPs. For example, simple replacement of AG with Mmj, a new photoconvertible FP, generated pcFluoppi, which allowed us to observe the intra- and inter-droplet exchange of liquid components for the analysis of PPI kinetics (Fig. 4).

We detected protein homo-dimerization with a single fusion construct containing PB1 and mAG1 (homoFluoppi). Many studies have highlighted the functional importance of protein homo-dimerization, and it is thought that in humans more than one-third of all enzymes function as homo-dimers^44^. However, some monomeric proteins can form non-physiological homo-dimers in crystals and monomer/dimer determinations can only be reliably conducted under physiological conditions^45^. Homo-dimerization of proteins in live cells is often detected by conventional FRET techniques that employ CFP and YFP^2^, or by a homo-FRET technique using YFP^46^. However, there are many systemic limitations to using FRET for PPI detection. The efficiency of homo-dimerization detection by CFP/YFP FRET is theoretically reduced to half after accounting for CFP-CFP and YFP-YFP associations. Also, homo-FRET detection requires a special device for anisotropy measurements. In either case, FRET-based approaches are severely limited by their low sensitivity for PPI detection.

HomoFluoppi signals provide physiologically relevant information for elucidating the quaternary structure of proteins under *in vivo* conditions. The dimeric model of active ERK2 (the best characterized mitogen-activated protein kinase) was proposed from the crystal structure of phosphorylated ERK2. Despite this commonly accepted view, there are contrary experimental data^47^. While *in vitro* experiments are criticized for their non-physiological conditions, most *in vivo* experiments fix cell samples or use homogenates prepared from hundreds of cells. Accordingly, when and where ERK2 homo-dimerizes and activates remains controversial. In this study, we used homoFluoppi to examine EGF-evoked ERK2 homo-dimerization in single living cells (Fig. 6c). We confirmed that dimerization depended on MEK kinase activity, and occurred in a pulsatile manner. Notably, there was heterogeneity in the timing and number of transient homoFluoppi signals in EGF-stimulated cells. Our results indicate that ERK2 homo-dimerization could only be precisely quantified in single living cells *in vivo* by the Fluoppi methodology.

We admit that Fluoppi is an artificial system and may not be able to monitor PPIs in a truly physiological context. Also, oligomerization of AG or PB1 may interfere with the function or localization of proteins to which they are fused. Nevertheless, we show that Fluoppi is useful for reliable detection of PPIs in live cells and can be used for studying PPI modulators and in principle the identification of novel PPIs. These properties make Fluoppi an ideal system for high content analysis and large scale drug screening studies. Altogether, Fluoppi may be used as a key technique in conjunction with existing methods to study quantitative protein interactions *in vivo*.

## Additional Information

**Accession codes** The sequences used in this study have been deposited in the DDBJ/EMBL/GenBank database under accession numbers LC087153 (AG-PB1), LC087154 (mAG1-PB1), LC087155 (PB1-FKBP), LC087156 (FRB-AG), LC087157 (PB1-p53), LC087158 (AG-MDM2), LC087159 (M13-PB1), LC087160 (CaM-KO), LC087161 (SmacNT-PB1), LC087162 (XIAP-AG), LC087163 (Mmj-PB1), LC087164 (Mmj-MDM2), LC087166 (PB1-BclXL-T2A-Mmj-BH3), LC087167 (PB1-p53-T2A-Mmj-MDM2), LC087168 (PB1-mAG1-FKBP12(F36V)), LC087169 (EGFP-PB1), LC087170 (mEGFP-PB1), LC091395 (FusionRed-PB1), LC087171 (FRB-mAG1), LC087171 (mPB1-FKBP), LC087173 (PB1-p50), LC087174 (AG-p65), LC087175 (AG-cRaf), LC087176 (PB1-KRas (WT)), LC087177 (PB1-KRas (S17N)), LC087178 (PB1-KRas (G12D)), LC087179 (Venus-AG), LC087180 (AG-Venus), LC087181 (PB1-AG), LC087182 (PB1-Venus), LC126313 (AG-p53). LC147071 (PB1-HRas), LC147072 (ERK2-mAG1-PB1).

**How to cite this article:** Watanabe, T. *et al*. Genetic visualization of protein interactions harnessing liquid phase transitions. *Sci. Rep.* **7**, 46380; doi: 10.1038/srep46380 (2017).

**Publisher's note:** Springer Nature remains neutral with regard to jurisdictional claims in published maps and institutional affiliations.

## Supplementary Material

Supplementary Video 1

Supplementary Video 2

Supplementary Video 3

Supplementary Video 4

Supplementary Video 5

Supplementary Video 6

Supplementary Video 7

Supplementary Video 8

Supplementary Video 9

Supplementary Video 10

Supplementary Video 11

Supplementary Information
