# Supplementary material for: Genetic visualization of protein interactions harnessing liquid phase transitions
Source: Sci Rep. 2017 Apr 13;7:46380. doi: 10.1038/srep46380 (PMC5390312; doi:10.1038/srep46380)
Supplement: Supplementary Information [file srep46380-s12.pdf]

SUPPLEMENTARY INFORMATION

Genetic Visualization of Protein Interactions Harnessing Liquid Phase Transitions

Taku Watanabe, Tatsuya Seki, Takashi Fukano, Asako Sakaue-Sawano, Satoshi Karasawa, Misaki Kubota, Hiroshi Kurokawa, Ken Inoue, Junichi Akatsuka, and Atsushi Miyawaki.

ONLINE METHODS

Supplementary Figure 1 | Fluorescence distribution in cytosol when PB1 was fused to AG or mAG1.

Supplementary Figure 2 | Validation and application of Fluoppi using PB1/AG tags.

Supplementary Figure 3 | An HCA approach to evaluation of drug-induced PPI modulation.

Supplementary Figure 4 | A new photoconvertible FP, Momiji (Mmj), and its application to Fluoppi (pcFluoppi).

Supplementary Figure 5 | PB1 concatemers required for generation or growth of Fluoppi puncta.

Supplementary Table 1 | PPIs quantified (characterized) in the present study (main figures).

Supplementary Table 2 | Comparison of Fluoppi properties with other FP-based PPI-detecting methods.

Supplementary Video 1 | Rapamycin-induced association between FRB and FKBP.

Supplementary Video 2 | Frequent fusion of fluorescent puncta.

Supplementary Video 3 | Nutlin-3-induced dissociation of p53/MDM2 complex.

Supplementary Video 4 | EGF-induced association between KRas and c-Raf.

Supplementary Video 5 | Histamine-induced oscillatory association between CaM and M13.

Supplementary Video 6 | AT-406-induced dissociation of Smac/XIAP complex.

Supplementary Video 7 | Time-lapse imaging of liquid-phase droplets.

Supplementary Video 8 | Phase separation due to association between HRas and cRaf.

Supplementary Video 9 | Pharmacological regulation of homo-dimerization of FKBP12(F36V).

Supplementary Video 10 | EGF-induced dimerization of ERK2.

Supplementary Video 11 | EGF-induced dimerization of ERK2.

References

ONLINE METHODS

Gene construction. All the plasmids used in this study were constructed from phmAG1-MNLinker or phmAG1-MCLinkler backbone (Medical Biological Laboratories, Amalgaam). The cDNA encoding 1–102 amino acids of human SQSTM (GenBank: NM_003900) was used as PB1. The following protein domains were used for PPI detection. mTOR: 2025–2114 amino acids of human MTOR (NM_004958), FKBP12: full length of human FKBP1A (NM_000801), p53: 1–70 amino acids of human TP53 (NM_000546), MDM2: 1–119 amino acids of human MDM2 (NM_002392), calmodulin: 3–149 amino acids of human CALM1 (NM_006888), M13 peptide: 566–591 amino acids of human MYLK2 (NM_033118), XIAP: 243–356 amino acids of human XIAP (NM_001167), SmacNT: 56–64 amino acids of human DIABLO (NM_019887), BclXL: 1–209 amino acids of human BCL2L1 (NM_138578), BH3: 103–127 amino acids of human BAD (NM_004322), HRas: 1–172 amino acids of human HRAS (NM_001130442), cRaf: 51–131 amino acids of human RAF1 (NM_002880), ERK2: full length of human MAPK. Site-directed mutations were introduced according to our protocols as described previously[46] to generate FKBP12(F36V) and PB1(D67A, D69R). The T2A sequence encoding GRGSLLTCGDVEENPGP was used for the equimolar expression of two constructs[47].

cDNA cloning. The stony coral *Scolymia vitiensis* was acquired from the sea off the Aka Island (Okinawa). Total RNA was isolated from the coral by guanidine thiocyanate extraction. Synthesis, amplification of the fragment of interest using degenerate primers, and generation of full-length cDNA were performed as previously described. The degenerate primers used were as follows: 5’-ATCAAGNTNWRYATGGAAGG-3’ and 5’- ACVGGDCCATYDGVAAGAAARTT-3’ (R = A or G; Y = C or T; V = A, C, or G; and D = A, G, or T). The cDNA encoding the protein-coding region was amplified using primers containing 5’-*Bam*HI and 3’-*Eco*RI sites. The restricted product was cloned in-frame into the *Bam*HI/*Eco*RI sites of $pRSET_B$ (Thermo Fisher Scientific) for bacterial expression. The 5’ end of the gene was modified by PCR to contain a Kozak consensus sequence (CCACCATG) after the *Bam*HI site to promote efficient expression in mammalian cells.

Protein expression. Recombinant Mmj protein with the polyhistidine tag at the N terminus was expressed in *Escherichia coli* (JM109 DE3) and purified as described[31].

Cell culture and transfection. HeLa, HEK293, Cos-7, U2OS, and CHO-K1 cells were purchased from ATCC. They were grown in Dulbecco’s modified Eagle’s medium (DMEM, Sigma) supplemented with 10% fetal bovine serum (FBS) and penicillin-streptomycin (Life Technologies). Cells were imaged 20–24 hours after transfection of plasmids with Polyfect (Qiagen) or Fugene HD (Promega). For the generation of stable cell lines of FRB-FKBP and p53-MDM2, transiently transfected cells were selected with G418 and hygromycin B followed by isolation of single clones by limited dilution.

Retrovirus construction and production. Retroviral vectors pQCXIP and pQCXIH (Clontech) were used to express XIAP-AG and SmacNT-PB1, respectively. Viral particles were generated in GP2-293 packaging cells (Clontech), and were used to transduce HEK293T cells to establish a stable cell line expressing both XIAP-AG and SmacNT-PB1. After selection with puromycin and hygromycin, single clones were isolated by limited dilution.

Wide-field imaging. Cells were grown on a 35-mm glass-bottom dish (IWAKI) or an 8-well glass-bottom chamber slide (Thermo Scientific). Imaging experiments were performed under an inverted microscope (Olympus IX71 with a standard 75-W xenon lamp, objective lenses, and a cooled charge-coupled device camera (ORCA-ER; Hamamatsu). The used objectives were ×20 (UPlanApo, N.A. = 0.7), ×40 (UPlanFLN, N.A. = 1.30), and ×60 (UPlanSApo, N.A. = 1.35). A filter unit (U-MGFPHQ; Olympus) composed of BP460-480HQ, BA495-540HQ, and DM485 was used for observing AG and non-photoconverted Mmj. A filter unit (U-MWU2; Olympus) composed of BP330-385, BA420, and DM400 was used for observing Hoechst33342. A filter unit composed of 580AF20, 600DRLP, and 645AF75 (Omega Optical) was used for observing photoconverted Mmj. A filter unit (FSET-KOHQ; Olympus) composed of BP520-540HQ, BA555-600HQ, and DM545HQ was used for observing KO. During

observation with EGF stimulation, cells were kept in DMEM containing 25 mM HEPES (pH 7.4), 10% FBS, and no phenol-red. A stage top incubator (MI-IBC; Tokai Hit) was used to control the temperature, CO_2 concentration, and humidity. Image acquisition and analysis were performed by using MetaMorph software (version 7.8.9.0) (Molecular Devices).

pcFluoppi. Photoconversion of Mmj was performed using light passing through a bandpass filter (BP330-385) and a homemade pinhole set at the position of the field stop. For measuring the red fluorescence from the photoconverted Mmj, clusters were segmented manually. Image acquisition and analysis were performed by using MetaMorph software (version 7.8.9.0) (Molecular Devices).

FRAP. Cells were grown on a 35-mm glass-bottom dish (IWAKI). FRAP experiments were performed using LSM 5 EXCITER (Carl Zeiss) with ×63 (Plan-Apochromat, N.A. = 1.4), an Argon gas laser (488 nm), and a bandpass filter (BP505-530). Image analyses were performed using ZEN black edition software (Carl Zeiss).

Time-lapse monitoring of punctate structures. Punctum intensity (P.I.) was measured as the intensity of fluorescence from identifiable puncta within a cell. Puncta were segmented automatically using the spot detector algorithm of the ICY open bioimage informatics platform[48]. Aspect ratio was measured automatically using the active contours algorithm (ICY).

Total internal reflection fluorescence microcopy (TIRFM) imaging. Cells were grown on a 35-mm glass-bottom dish (IWAKI). Imaging experiments were performed under an inverted microscope (Olympus IX71 with a standard 75-W xenon lamp) and a cooled charge-coupled device camera (ORCA-ER; Hamamatsu). The used objective was ×60 (ApoN, N.A. = 1.49). A filter unit (U-MGFPHQ; Olympus) composed of BP460-480HQ, BA495-540HQ, and DM485 was used for observing AG fluorescence. Image acquisition and analysis were performed by using MetaMorph software (version 7.8.9.0) (Molecular Devices).

Dose-response relationship studies (Nutlin-3) by high-content analysis. Stable transformant cells were seeded into poly-D-lysine black-wall 96-well plates (Corning) at a density of 40,000 cells/well and incubated for 20 hours at 37 °C in a 5% CO_2 atmosphere. After treatment with nutlin-3 for 30 minutes at room temperature, cells were fixed with 4% paraformaldehyde (PFA) for 10 minutes, and then stained with Hoechst33342 (Dojindo) for 30 minutes. Image acquisition was performed using IN Cell Analyzer 2200 (GE Healthcare). A 10× objective lens was used so that each field of view contained > 2,206 cells. For each concentration of nutlin-3, three wells were used. Image analysis was performed using IN Cell Investigator software (GE Healthcare). Green (AG) and blue (Hoechst33342) fluorescence images were used to monitor cytoplasmic puncta and nuclei, respectively (see **Fig. 3a**); they were segmented automatically using the granular and nuclear segmentation algorithms, respectively. P.I. values were calculated by dividing the total green fluorescence by the number of nuclei (cells). The averaged P.I. values were normalized to that at the lowest concentration and plotted (**Fig. 3b**). Curve fitting was performed using KaleidaGraph (Synergy Software).

Dose-response relationship analysis (Nutlin-3) using a plate reader. Stable transformant cells were seeded onto poly-D-lysine black-wall 96-well plates (Corning) at a density of 20,000 cells/well and incubated for 20 hours at 37 °C in a 5% CO_2 atmosphere. After treatment with nutlin-3 for 30 minutes at room temperature, cells were fixed and permeabilized with 0.75% PFA and 2% Triton-X 100 for 15 minutes, and then stained with Hoechst33342 (Dojindo) for 30 minutes. Representative images (**Fig. 3c**) were acquired using IX71 (Olympus). Fluorescence intensities of AG (green) and Hoechst33342 (blue) were measured using a Wallac Arvo HTS 1420 Multilabel Counter (Perkin-Elmer). For each concentration of nutlin-3, three wells were used. P.I. values were simply calculated by dividing the green signal intensity by the blue signal intensity. The averaged P.I. values were normalized to that at the lowest concentration and plotted (**Fig. 3d**). Curve fitting was performed using KaleidaGraph (Synergy Software).

Dose-response relationship studies (AT-406) by high-content analysis. Stable transformant cells were seeded onto poly-D-lysine black-wall 96-well plates (Corning)

at a density of 40,000 cells/well and incubated for 20 hours at 37 °C in a 5% CO_2 atmosphere. After treatment with AT-406 for 15 minutes at room temperature, cells were fixed with 4% PFA for 10 minutes, and then stained with Hoechst33342 (Dojindo) for 30 minutes. Image acquisition was performed using Cell Voyager CV7000 (Yokogawa Electric Corporation). A 20× objective lens was used so that each field of view contained > 2,679 cells. For each concentration of AT-406, four wells were used. Image analysis was performed using Cell Voyager analytical software (Yokogawa Electric Corporation). Green (AG) and blue (Hoechst33342) fluorescence images were used to monitor cytoplasmic puncta and nuclei, respectively (see **Fig. 3f**); they were segmented automatically using the granular and nuclear segmentation algorithms, respectively. P.I. values were calculated by dividing the total green fluorescence by the number of nuclei (cells). The averaged P.I. values were normalized to that at the lowest concentration and plotted (**Fig. 3g**). Curve fitting was performed using KaleidaGraph (Synergy Software).

Chemical compounds. Rapamycin and nutlin-3 were purchased from Merck Millipore. AT-406 and LCL-161 were purchased from Active Biochemicals. B/B Homodimerizer (HD) and B/B Washout Ligand (WL) were purchased from Takara-Bio.

Supplementary Figure 1 | Fluorescence distribution in cytosol when PB1 was fused to AG or mAG1.

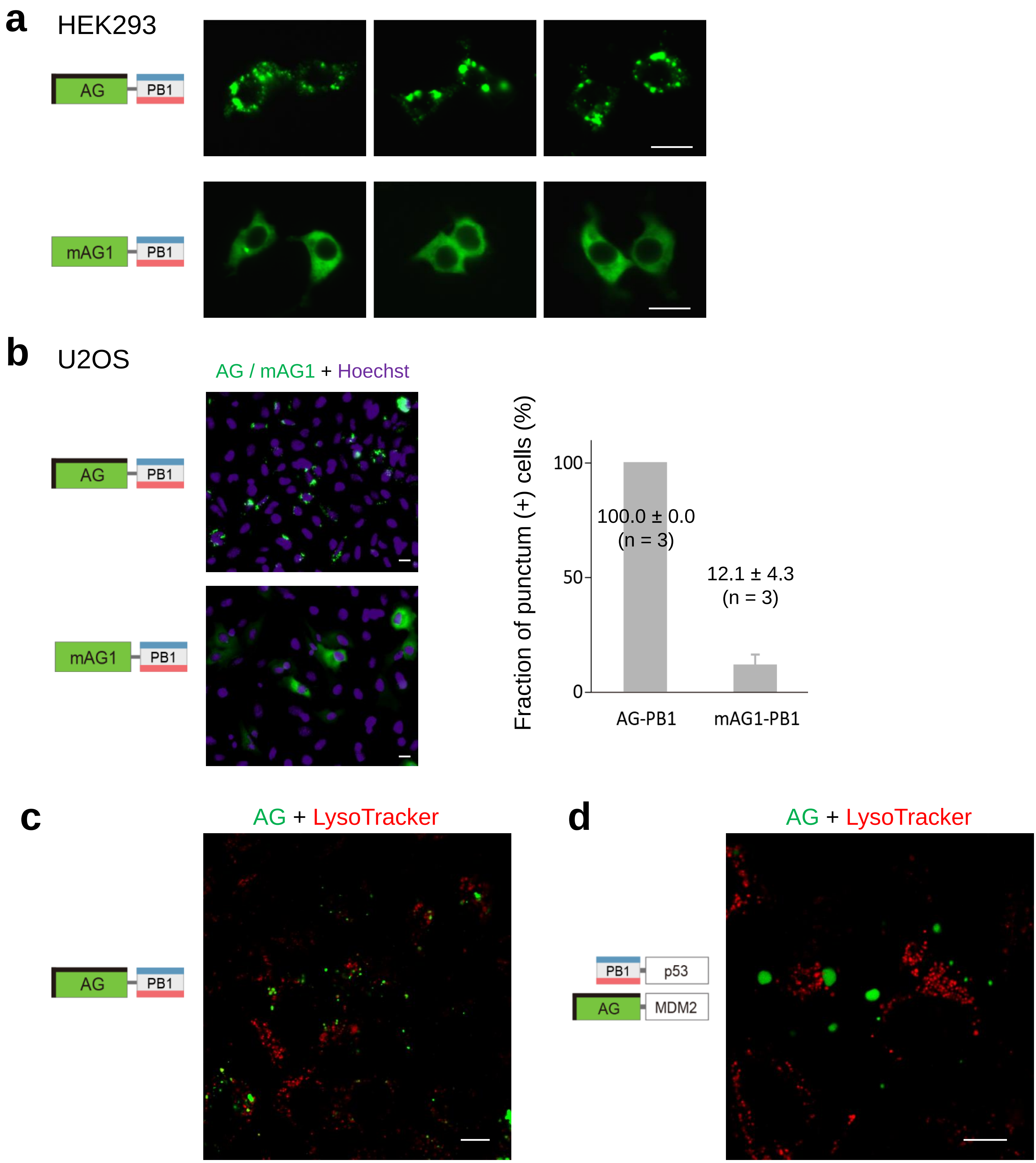

(**a**) Representative fluorescence images of HEK293 cells expressing AG-PB1 (upper) and mAG1-PB1 (lower). Images were acquired one day post-transfection. Scale bars, 10 μm. (**b**) Representative fluorescence images of U2OS cells expressing AG-PB1 (upper) and mAG1-PB1 (lower). Images were acquired one day post-transfection after nuclear staining with Hoechst33342. The fraction of cluster-carrying cells in examined cells for AG-PB1 or mAG1-PB1 was calculated. Approximately 20 cells were examined in triplicate, and data are shown as mean ± SEM. Scale bars, 10 μm. (**c, d**) Representative fluorescence images of HeLa cells expressing AG-PB1 (**c**) and PB1-p53/AG-MDM2 (**d**). Images were acquired one day post-transfection and after incubation with 50 nM LysoTracker Red DND-99 (L7528) in HBSS at 37 ºC for 30 min. Fluorescent puncta were well separated from lysosomes. Scale bars, 10 μm.

Supplementary Figure 2 | Validation and application of Fluoppi using PB1/AG tags.

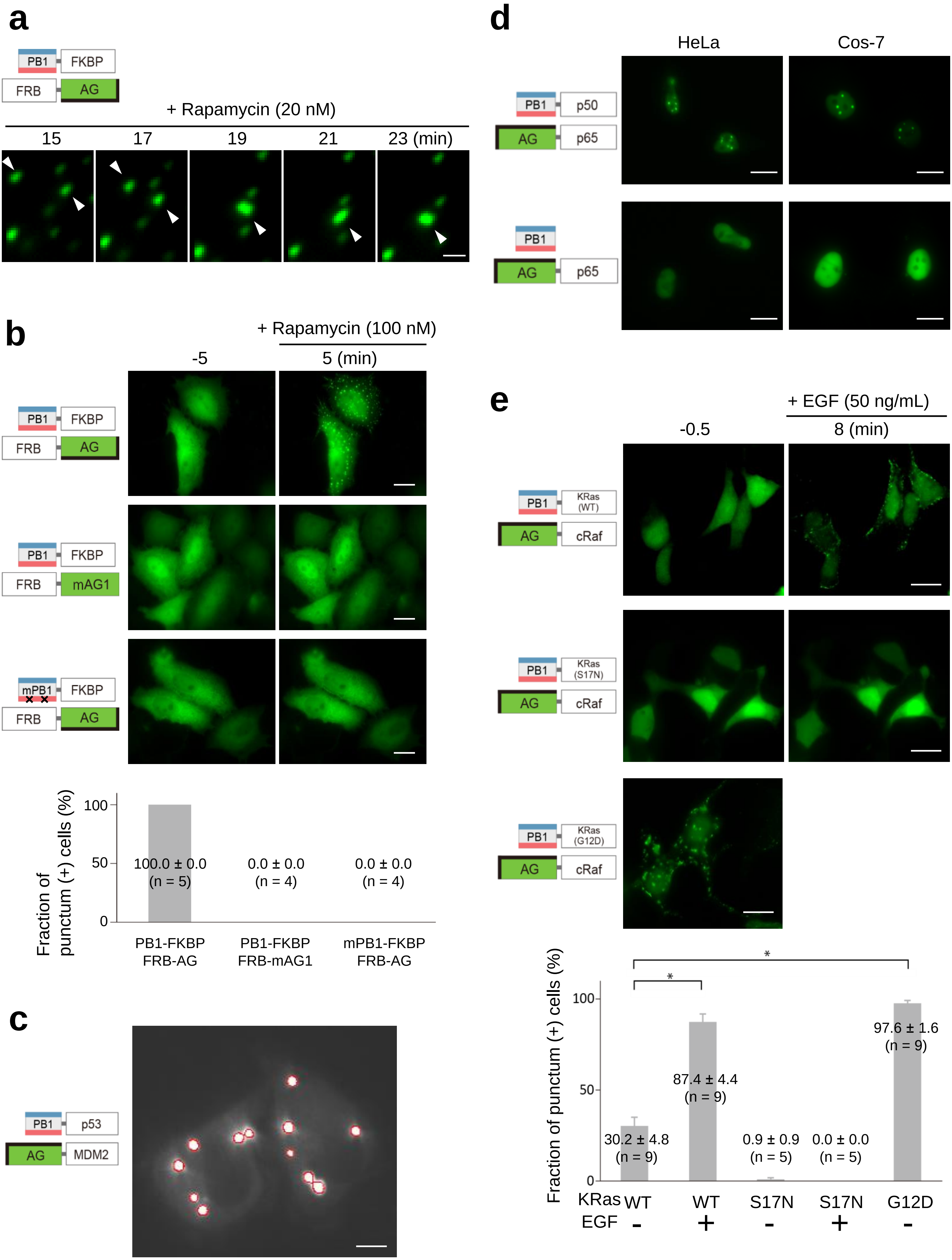

(**a**) Temporal montage of rapamycin-induced development of fluorescent puncta in HeLa cells co-expressing PB1-FKBP and FRB-AG. Arrowheads indicate two puncta that collided with each other and then fused. Scale bar, 1 μm. (**b**) Visualization of rapamycin-induced association between FRB and FKBP in HeLa cells required the homo-oligomerizing capability of both AG and PB1. One day post-transfection of PB1-FKBP/FRB-AG (top), PB1-FKBP/FRB-mAG1 (middle), and mPB1 (monomeric PB1 with two mutations: D67A and D69R)-FKBP/FRB-AG (bottom), cells were imaged before (-5 min) and after (5 min) the addition of 100 nM rapamycin. The fraction of punctum-carrying cells in examined cells for the above three transfections was calculated. Data are shown as mean ± SEM (n = 4 or 5). Scale bars, 10 μm. (**c**) Segmentation of fluorescent puncta for calculation of P.I. Segmentation was performed using 'Spot detector' of the ICY open bioimage informatics platform. Scale bar, 10 μm. (**d**) Application of Fluoppi to PPI occurring inside the nucleus. Representative fluorescence images of HeLa (left) and Cos-7 (right) cells expressing PB1-p50/AG-p65 (upper) and PB1/AG-p65 (lower). Images were acquired one day post-transfection. Punctum formation was observed inside the nucleus depending on the association between p50 and p65. Scale bars, 10 μm. (**e**) Application of Fluoppi to PPI occurring beneath the plasma membrane. Representative fluorescence images of HEK293 cells expressing PB1-KRas(wild type)/AG-cRaf (upper), PB1-KRas(S17N)/AG-cRaf (middle), and PB1-KRas(G12D)/AG-cRaf (bottom). Cells were imaged before (-0.5 min) and after (8 min) the addition of 50 ng/mL epidermal growth factor (EGF). Punctum formation was observed on the plasma membrane depending on the EGF-induced association between KRas and cRaf. The fraction of punctum-carrying cells in examined cells under the five conditions was calculated. Approximately 20 cells were observed in quintuplicate, and data are shown as mean ± s.e.m (n = 5 or 9). Statistical significance (*$p < 1E-9$) was examined by Bonferoni's multiple comparison test. Scale bars, 10 μm.

Supplementary Figure 3 | An HCA approach to evaluation of drug-induced PPI modulation.

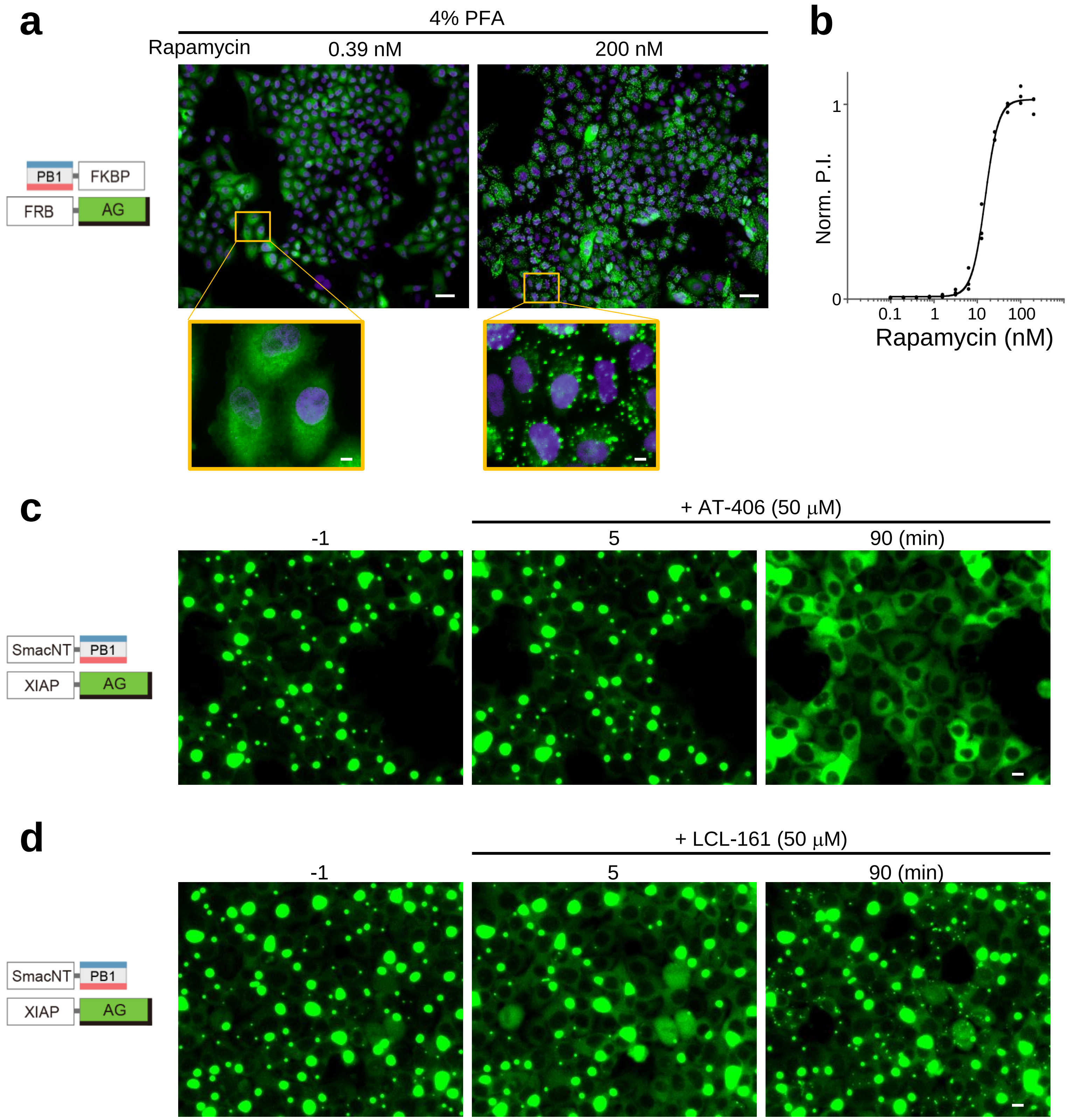

(**a, b**) Rapamycin-induced association of FKBP/FRB complex. HeLa cells stably co-expressing PB1-FKBP and FRB-AG were used. After treatment of various concentrations of rapamycin for 30 min, cells were fixed with 4% PFA for 10 min and their nuclei were stained with Hoechst33342. (**a**) Domain structures of transfected constructs and two representative images of cells treated with 0.39 and 200 nM rapamycin. (**b**) Dose (rapamycin concentration)-response (normalized P.I.) curve. Both puncta and nuclei were segmented automatically. P.I. was obtained by dividing the total AG (green) fluorescence from all the puncta by the total number of nuclei in each field of view. Normalized to the P.I. value at the highest concentration.
Scale bars, 100 μm. 10 μm (in magnified boxes). (**c, d**) Monitoring dissociation of Smac/XIAP complex in HEK293 cells stably co-expressing SmacNT-PB1 and XIAP-AG after the addition of AT-406 (**c**) and LCL-161 (**d**). Domain structures of transfected constructs are illustrated (leftmost). The time point of image acquisition relative to drug administration is indicated above each image. The effect of 50 μM AT-406 was slow but strong enough to eliminate Fluoppi puncta completely at 90 min. In contrast, the effect of 50 μM LCL-161 was fast but rather moderate and gradually attenuated. Scale bars, 10 μm.

Supplementary Figure 4 | A new photoconvertible FP, Momiji (Mmj), and its application to Fluoppi (pcFluoppi).

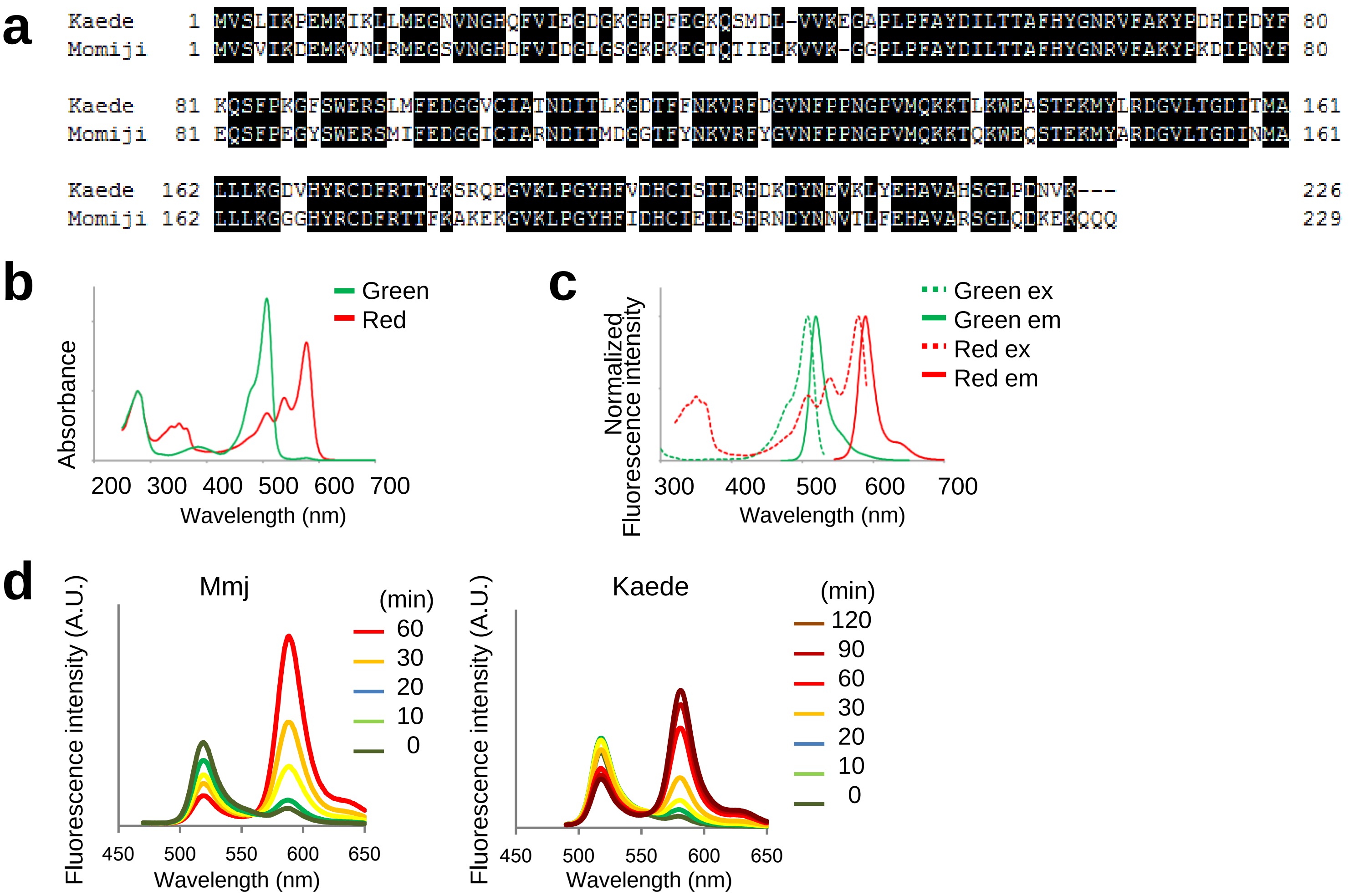

e

Momiji (Mmj) (229 amino acids)	Ex / Em (nm)	Molar Extinction Coefficient ($M^{-1} \cdot cm^{-1}$)	Fluorescence Quantum Yield	pKa
Green	508 / 518	102,250 (508 nm)	0.43	5.8
Red	578 / 588	76,950 (578 nm)	0.51	6.5

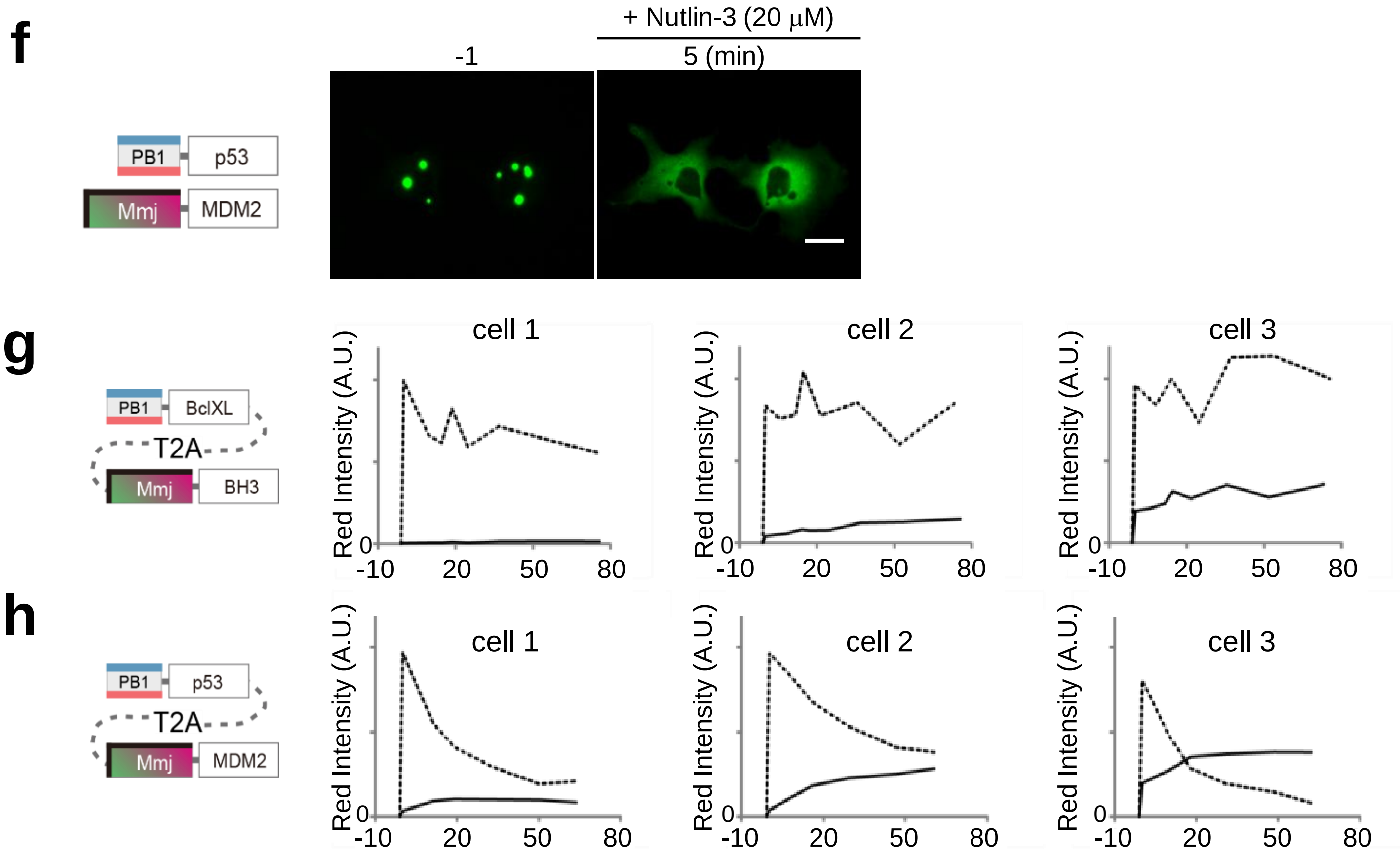

(**a**) Amino acid sequence (single-letter code) alignment of Momiji (Mmj) with Kaede. (**b**) Absorption spectra of unconverted (green) and converted (red) states of Momiji. (**c**) Excitation (broken line) and emission (solid line) spectra of unconverted (green) and converted (red) states of Momiji. The spectra were normalized to their highest values. (**d**) Emission spectra of Momiji (Mmj) and Kaede on excitation at 365 nm during the green-to-red conversion that was executed by continuous illumination at 365 nm. (**e**) Properties of green and red states of Momiji. (**f**) Nutlin-3-induced dissociation of p53/MDM2 complex. One day post-cotransfection of PB1-p53 and Mmj-MDM2, cells were treated with 20 μM nutlin-3. Fluorescence images were acquired before (-1 min) and after (5 min) the addition of nutlin-3. Scale bars, 10 μm. (**g**) Similar data on the dissociation of the BcLXL/BH3 complex were obtained from three other cells. (**h**) Similar data on the dissociation of the p53/MDM2 complex were obtained from three other cells.

Supplementary Figure 5 | PB1 concatemers required for generation or growth of Fluoppi puncta.

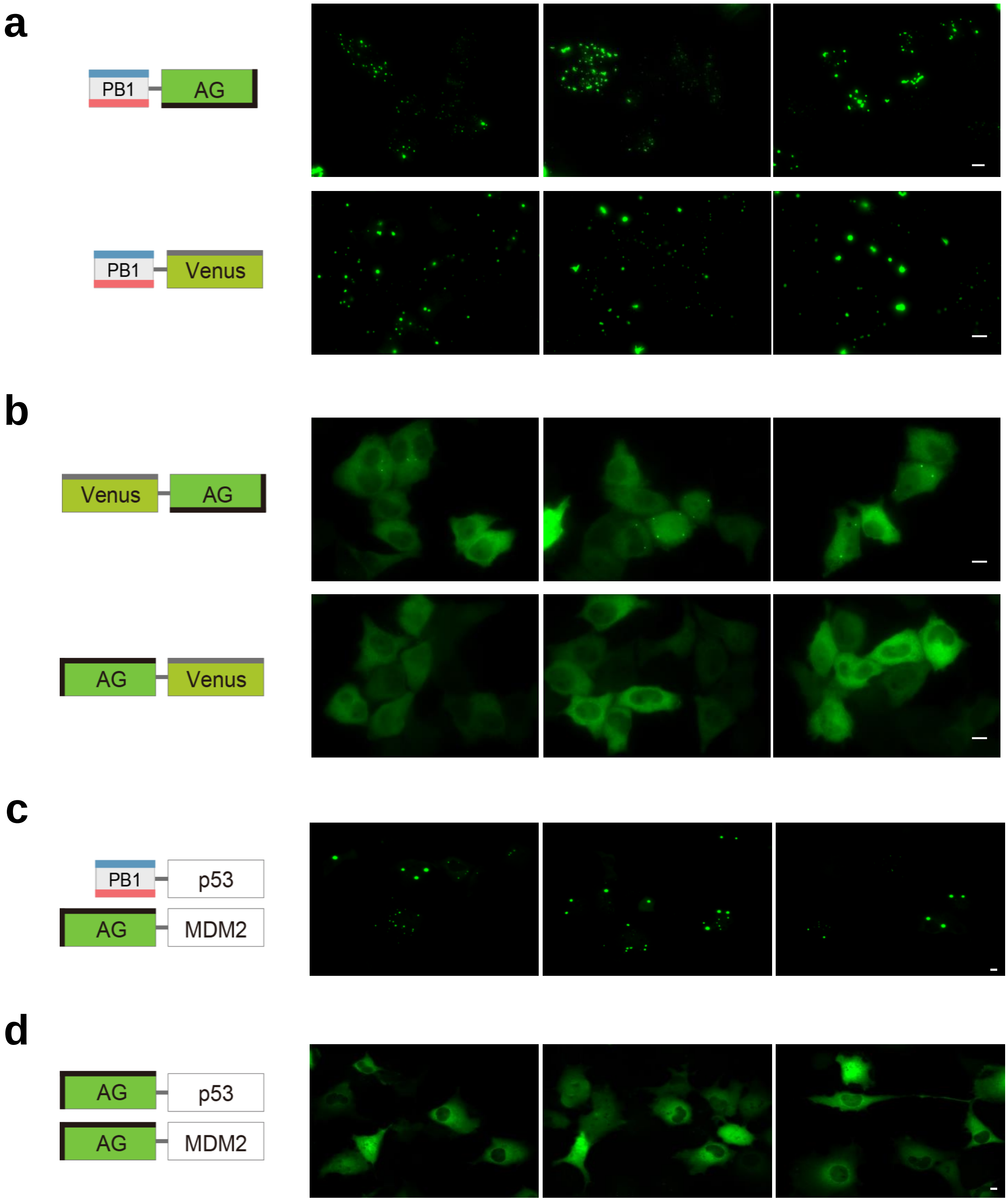

(**a**) PB1 was fused to AG or Venus to form PB1-AG (upper) and PB1-Venus (lower), respectively. Both constructs generated large fluorescent puncta. Related to Figure 1, which shows image data of AG-PB1. (**b**) Two different homo-oligomerizing FPs used in (**a**) were fused covalently. Venus-AG (upper) and AG-Venus (lower). Neither of them generated fluorescent puncta. (**c**) Co-transfection of PB1-p53 and AG-MDM2 generated large fluorescent puncta. Similar to Figure 2C. (**d**) Co-transfection of AG-p53 and AG-MDM2 generated no puncta. (**a–d**) Scale bars, 10 μm.

Supplementary Table 1 | PPIs quantified (characterized) in the present study (main figures).

	Figure		Construct	Event	Stimulation	Quantification			
						Segmentation		Punctum intensity	Graph
						Punctum	Cell (Nuc)		
Fluoppi	2	c	PB1 - p53 : AG - MDM2	dissociation	Nutlin-3	automatic	manual	AG fluorescence / cell no.	Temporal Profile
		d	M13 - PB1 : CaM - KO	oscillatory association	Histamine			KO fluorescence / cell no.	Temporal Profile
	3	b	PB1 - p53 : AG - MDM2	dissociation	Nutlin-3	automatic	automatic	AG fluorescence / cell no.	Dose-Response Curve
		d	PB1 - p53 : AG - MDM2	dissociation	Nutlin-3	-	-	AG fluorescence / Hoechst fluorescence	Dose-Response Curve
		e	SmacNT - PB1 : XIAP - AG	dissociation	AT-406	automatic	manual	AG fluorescence / cell no.	Temporal Profile
		f	SmacNT - PB1 : XIAP - AG	dissociation	AT-406	automatic	automatic	AG fluorescence / cell no.	Dose-Response Curve
pcFluoppi	4	b	PB1 - p53 : Mmj - MDM2	fast exchange	light	-	-	Mmj (red) fluorescence / region	
		c	PB1 - BclXL : Mmj - BH3	slow exchange					
		d	PB1 - p53 : Mmj - MDM2	fast exchange					
homoFluoppi	6	b	PB1 - mAG1 - FKBP12(F36V)	association dissociation	B/B Homodimerizer B/B Washout Ligand	automatic	manual	mAG1 fluorescence / cell no.	Temporal Profile
		c	ERK2 - mAG1 - PB1	pulsatile association	EGF	automatic	manual	mAG1 fluorescence / cell no.	Temporal Profile

Supplementary Table 2 | Comparison of Fluoppi properties with other FP-based PPI-detecting methods.

	Redistribution							Tag proximity		
	Phase transition	Cluster formation	Translocation to platforms					RET	PCA	dimer formation
Properties	Fluoppi	InCell SMART-i	F2H (Lac operator)	F3H (Lac oparator)	F3H (MBD/Lamin/Centrin)	FLS	GRIP	FRET	BiFC	ddFP
Reversibility (Dissociation)	Yes	N.T.	Yes	Yes	Yes	N.T.	Yes	Yes	No	Yes
PPI Kinetics (Kon, Koff)	Yes	?	No	No	No/ Yes (Lamin)	?	N.T.	No	No	No
Foci formation by chemicals	No	Yes (Rapamycin)	No	No	No	No	Yes (RS25344)	—	—	—
Dynamic range	High	High	Modest[d]	Modest[d]	Modest[d]/ High (MBD)	High[g]	Modest[d]	Low	High	Modest ~ High
Simplicity	Yes	No[b, c]	No[e]	No[c, e]	No[c]	?[g]	No[h]	No[i]	Yes	Yes
Compartment	JM/Cyto/Nuc	JM/Cyto/Nuc	Nuc	Nuc	Cyto/Nuc	Cyto	Cyto	Anywhere	Anywhere	Anywhere
Homodimer detection	Efficient[a]	Possible	Possible	Possible	Possible	Possible	Possible	Possible	Possible	Possible
Wide applicability	Yes	Yes	Yes	Yes	N.T.[f]	Yes	N.T.[f]	Yes	Yes	Yes
Fixation resistance	Yes	Yes	Yes	Yes	Yes	Yes	Yes	No	Yes	N.T.
HCA (Image based)	Yes	N.T.	Yes	N.T.	N.T.	N.T.	Yes	Yes	Yes	N.T.
HTS (Detection by PMT)	Yes	N.T.	N.T.	N.T.	N.T.	N.T.	N.T.	Yes	Yes	N.T.
References	This report	9, 10	6, 8, 49–55	7, 56	7	41, 57	58	2, 26, 59, 60	3, 61	4, 5

a, Single fusions are sufficient.
b, Requires Rapamycin.
c, Requires 3 or more gene transfection.
d, High background noise or limited dynamic range.
e, Requires stable cell lines which possess the Lac operator integrated genome.
f, Only p53/MDM2 reported.
g, Accompanied by immunostaining in mammalian cells.
h, Requires RS25344.
i, Requires expertise.

Bioluminescent methods, such as bioluminescence resonance energy transfer, are not covered here.

N.T., Not tested.
RET, Resonance energy transfer.
PCA, Protein complementation assay.
F2H, Fluorescent two hybrid.
F3H, Fluorescent three hybrid strategy using genome-integrated Lac operator array.
F3H, Fluorescent three hybrid strategy using MBD, Lamin or Centrin platform.
FLS, Factory-like structures.
GRIP, Green fluorescent protein-assisted readout for interacting proteins.
BiFC, Bimolecular fluorescence complementation.
HCA, High content analysis.
HTS, High throughput screening.
PMT, Photomultiplier tube.
JM, Juxta Membrane ; Cyto, Cytosol; Nuc, Nucleus.

Supplementary Video 1 | Rapamycin-induced association between FRB and FKBP.
Visualization of rapamycin-induced association between FRB and FKBP in HeLa cells stably coexpressing PB1-FKBP and FRB-AG. Imaged every approximately 10 seconds. Observation time (min:sec). Rapamycin (20 nM) was added at t = 00:00. **Fig. 2b** is a temporal montage made from this movie.

Supplementary Video 2 | Frequent fusion of fluorescent puncta.
Expanded view of a cell shown in **Supplementary Video 1**. Observation time (min:sec).

Supplementary Video 3 | Nutlin-3-induced dissociation of p53/MDM2 complex.
Visualization of nutlin-3-induced dissociation of p53/MDM2 complex in two HeLa cells (cell 1 and cell 2) that transiently co-expressed PB1-p53 and AG-MDM2. Imaged every approximately 10 seconds. Observation time (min:sec). Nutlin-3 (20 μM) was added at t = 00:00. **Fig. 2c** is a temporal montage made from this movie.

Supplementary Video 4 | EGF-induced association between KRas and c-Raf.
Visualization of EGF-induced association between KRas and c-Raf in Hela cells coexpressing PB1-KRas and AG-cRaf. Imaged every approximately 10 seconds. Observation time (min:sec). EGF (50 ng/mL) was added at t = 00:00. **Supplementary Fig. 2e** (upper) is a temporal montage made from this movie.

Supplementary Video 5 | Histamine-induced oscillatory association between CaM and M13.
Visualization of histamine-induced oscillatory association between CaM and M13 in a HeLa cell that co-expressed M13-PB1 and CaM-KO. The image acquisition interval was 1.5 seconds. Observation time (seconds) is indicated at the lower right. Histamine was added at t = 0. **Fig. 2d** is a temporal profile made from this movie.

Supplementary Video 6 | AT-406-induced dissociation of Smac/XIAP complex.
Visualization of AT-406-induced dissociation of Smac/XIAP complex in HEK293 cells stably coexpressing SmacNT-PB1 and XIAP-AG. Imaged every approximately 30 seconds. Observation time (min:sec). AT-406 (25 μM) was added at t = 00:00. **Fig. 3e** is a temporal montage made from this movie.

Supplementary Video 7 | Time-lapse imaging of liquid-phase droplets.
Observation of CHO-K1 cells stably coexpressing PB1-p53 and AG-MDM2. Imaged every 15 minutes. Observation time (hr:min). **Fig. 5c** is a temporal montage made from this movie.

Supplementary Video 8 | Phase separation due to association between HRas and cRaf. Visualization of 2D phase separation on the plasma membrane. Micron-sized structures appeared depending on the association between HRas and cRaf induced by EGF stimulation and PB1polymerization. Cos-7 cells co-expressing PB1-HRas and AG-cRaf (left) and mPB1-HRas and AG-cRaf (right) were time-lapse imaged by TIRF microscopy. Imaged every approximately 10 seconds. Observation time (min:sec). EGF (50 ng/mL) was added at t = 00:00. **Fig. 5d** and **Fig. 5e** show representative images made from these two movies (left and right, respectively).

Supplementary Video 9 | Pharmacological regulation of homo-dimerization of FKBP12(F36V).
Visualization of homo-dimerization of FKBP12(F36V) induced by HD (500 nM) and then disrupted by WL (1 μM) in HEK cells expressing PB1-mAG1-FKBP12(F36V). Imaged every 10 min. Observation time (min:sec). HD was added at 29:59. Washing was done at 180:06. WL was added at 204:19. **Fig. 6b** is a temporal montage made from this movie.

Supplementary Video 10 | EGF-induced dimerization of ERK2.
Visualization of EGF-induced dimerization of ERK2 in a Cos-7 cell expressing ERK2-mAG1-PB1. Imaged every approximately 10 seconds. Observation time (min:sec). EGF (50 ng/mL) was added at t = 00:00. **Fig. 6c** (top) is a temporal montage made from this movie.

Supplementary Video 11 | EGF-induced dimerization of ERK2.
Visualization of EGF-induced dimerization of ERK2 in a Cos-7 cell expressing ERK2-mAG1-PB1. Imaged every approximately 10 seconds. Observation time (min:sec). EGF (50 ng/mL) was added at t = 00:00. **Fig. 6c** (middle) is a temporal montage made from this movie.

References

46. Sawano, A. & Miyawaki, A. Directed evolution of green fluorescent protein by a new versatile PCR strategy for site-directed and semi-random mutagenesis. *Nucleic Acids Res.* **28**, E78 (2000).

47. Kim, J.H. *et al.* High cleavage efficiency of a 2A peptide derived from porcine teschovirus-1 in human cell lines, zebrafish and mice. *PloS ONE* **6**, e18556 (2011).

48. de Chaumont, F. *et al.* Icy: an open bioimage informatics platform for extended reproducible research. *Nat. Methods* **9**, 690–696 (2012).

49. Meilinger, D. *et al.* Np95 interacts with de novo DNA methyltransferases, Dnmt3a and Dnmt3b, and mediates epigenetic silencing of the viral CMV promoter in embryonic stem cells. *EMBO Rep.* **11**, 1259–1264 (2009).

50. Mortusewicz, O., Fouquerel, E., Amé, J.C., Leonhardt, H. & Schreiber, V. PARG is recruited to DNA damage sites through poly(ADP-ribose)- and PCNA-dependent mechanisms. *Nucleic Acids Res.* **12**, 5045–5056 (2011).

51. Zolghadr, K., Rothbauer, U. & Leonhardt, H. The fluorescent two-hybrid (F2H) assay for direct analysis of protein-protein interactions in living cells. *Methods Mol. Biol.* **812**, 275–282 (2012).

52. Brown, C.J. *et al.* Stapled peptides with improved potency and specificity that activate p53. *ACS Chem. Biol.* **8,** 506–512 (2013).

53. Wei, S.J. *et al.* Inhibition of nutlin-resistant HDM2 mutants by stapled peptides. *PloS ONE* **8**, e81068 (2013).

54. Wei, S.J. *et al.* In vitro selection of mutant HDM2 resistant to Nutlin inhibition. *PloS ONE* **8**, e62564 (2013).

55. Wolf, A. et al. The polyserine domain of the lysyl-5 hydroxylase Jmjd6 mediates subnuclear localization. *Biochem. J.* **453**, 357–370 (2013).

56. Dambacher, S. *et al.* CENP-C facilitates the recruitment of M18BP1 to centromeric chromatin. *Nucleus* **3**, 101–110 (2012).

57. Schmitz, A.M., Morrison, M.F., Agunwamba, A.O., Nibert, M.L. & Lesser, C.F. Protein interaction platforms: visualization of interacting proteins in yeast. *Nat. Methods* **7**, 500-502 (2009).

58. Lundholt, B.K., Heydorn, A., Bjorn, S.P. & Praestegaard, M. A simple cell-based HTS assay system to screen for inhibitors of p53-Hdm2 protein-protein interactions. *Assay Drug Dev. Technol.* **4**, 679–688 (2006).

59. Seth, A., Otomo, T., Yin, H.L. & Rosen, M.K. Rational design of genetically encoded fluorescence resonance energy transfer-based sensors of cellular Cdc42 signaling. *Biochemistry* **42**, 3997–4008 (2003).

60. Tsien, R.Y. Nobel lecture: constructing and exploiting the fluorescent protein paintbox. *Integr. Biol.* **2**, 77–93 (2010).

61. Shekhawat, S.S. & Ghosh, I. Split-protein systems: beyond binary protein-protein interactions. *Curr. Opin. Chem. Biol.* **15**, 789–797 (2011).
